# Development of brain atlases for early-to-middle adolescent collision-sport athletes

**DOI:** 10.1038/s41598-021-85518-6

**Published:** 2021-03-19

**Authors:** Yukai Zou, Wenbin Zhu, Ho-Ching Yang, Ikbeom Jang, Nicole L. Vike, Diana O. Svaldi, Trey E. Shenk, Victoria N. Poole, Evan L. Breedlove, Gregory G. Tamer, Larry J. Leverenz, Ulrike Dydak, Eric A. Nauman, Yunjie Tong, Thomas M. Talavage, Joseph V. Rispoli

**Affiliations:** 1 Weldon School of Biomedical Engineering, Purdue University, West Lafayette, IN 47907 USA; 2 College of Veterinary Medicine, Purdue University, West Lafayette, IN 47907 USA; 3 Department of Statistics, Purdue University, West Lafayette, IN 47907 USA; 4 School of Electrical and Computer Engineering, Purdue University, West Lafayette, IN 47907 USA; 5 Department of Basic Medical Sciences, Purdue University, West Lafayette, IN 47907 USA; 6 School of Mechanical Engineering, Purdue University, West Lafayette, IN 47907 USA; 7 Department of Health and Kinesiology, Purdue University, West Lafayette, IN 47907 USA; 8 School of Health Sciences, Purdue University, West Lafayette, IN 47907 USA

**Keywords:** Computational neuroscience, Data integration, Databases, Image processing, Statistical methods

## Abstract

Human brains develop across the life span and largely vary in morphology. Adolescent collision-sport athletes undergo repetitive head impacts over years of practices and competitions, and therefore may exhibit a neuroanatomical trajectory different from healthy adolescents in general. However, an unbiased brain atlas targeting these individuals does not exist. Although standardized brain atlases facilitate spatial normalization and voxel-wise analysis at the group level, when the underlying neuroanatomy does not represent the study population, greater biases and errors can be introduced during spatial normalization, confounding subsequent voxel-wise analysis and statistical findings. In this work, targeting early-to-middle adolescent (EMA, ages 13–19) collision-sport athletes, we developed population-specific brain atlases that include templates (T1-weighted and diffusion tensor magnetic resonance imaging) and semantic labels (cortical and white matter parcellations). Compared to standardized adult or age-appropriate templates, our templates better characterized the neuroanatomy of the EMA collision-sport athletes, reduced biases introduced during spatial normalization, and exhibited higher sensitivity in diffusion tensor imaging analysis. In summary, these results suggest the population-specific brain atlases are more appropriate towards reproducible and meaningful statistical results, which better clarify mechanisms of traumatic brain injury and monitor brain health for EMA collision-sport athletes.

## Introduction

Adolescent collision-sport (e.g., American football and soccer) athletes bear high risk of mild traumatic brain injury (mTBI), a complex pathophysiological process that can arise from either single or repetitive head acceleration events^1–4^. The lack of sensitive biomarkers hinders the development of preventive strategies, allowing this vulnerable population to continue participating at greater risk. Multi-modal magnetic resonance imaging (MRI) can non-invasively characterize the structure and function of the human brain in healthy and disease states, thus showing promise for prospective screening and early detection of mTBI in adolescent athletes. Nevertheless, one of the critical steps in MRI processing is to spatially normalize brain images to a stereotaxic atlas, i.e., a coordinate reference system for neuroimaging studies. When the spatial normalization onto an atlas has poor accuracy, voxel-based analysis exhibits low sensitivity in detecting differences at the group level^5, 6^. Therefore, it is vital to ensure most of the anatomical identities pertinent to adolescent athletes are retained during spatial normalization.

In general, human brain atlases are either standardized or population-specific; each comes with a set of templates (representative spatial maps) and labels (parcellated regions). The two standardized brain atlases long established and well known by the neuroimaging community come from Talairach and the Montreal Neurological Institute^7^. The Talairach atlas is derived from the dissection of one hemisphere of the brain from a 60-year-old French woman^8^, whereas the ICBM152 template is derived from T1 scans of 152 subjects aged 18.5–43.5, averaged together after high-dimensional linear and nonlinear registration into the Talairach space^9^. In the same space as ICBM152, FMRIB58 is a standardized diffusion tensor imaging (DTI) template derived from 58 high-resolution volumes of fractional anisotropy (FA) from healthy male and female adults aged 20–50 (FMRIB, Oxford, UK). Other popular standardized human brain atlases include Brainnetome^10^, IIT^11^, SRI24^12^, etc. Recently, two systematic evaluations of DTI templates showed that the IIT standard template outperformed population-specific DTI templates^11, 13^, but the findings were based on healthy adults and may not generalize in younger populations. Many scientific publications pointed out the age-related changes in volumes of gray and white matter^14–19^. Although there are several age-specific atlases for adolescents^20–25^, the number is limited compared to adult atlases^14^.

The existing brain atlases are handy tools for various types of neuroimaging analyses, but considering the various pathological conditions and the developing nature of human brain, they often are not best suited for studying specific populations. In a multiple sclerosis population, Van Hecke et al.^26^ showed that choosing a non-specific template can negatively impact the final results of tract-based spatial statistics (TBSS)^27^, one of the standard approaches for DTI analysis^5, 28^. Using both simulated and real DTI data, Van Hecke et al. observed that a population-specific DTI template resulted in more reliable voxel-based analysis, as well as higher sensitivity and specificity of detecting DTI changes, compared to the standardized template^26^. However, developing a study-specific template from a large population is time consuming, computationally inefficient^28^, and may result in suboptimal quality^11, 26^, making the use of existing brain atlases a more pragmatic option for the time being.

To date, an unbiased brain atlas targeting adolescent collision-sport athletes does not exist, to the best of our knowledge. DTI literature of sports-related mTBI and repetitive head impacts in adolescents either manually defined their own regions of interest (ROIs)^29–31^, or more often did not employ a population-specific template to spatially normalize each individual brain image^32–44^. This may confound statistical analyses and contribute to varied DTI findings that make it difficult to interpret axonal pathology^45^. It is critical for studies of mTBI and repetitive head impacts, especially for adolescent collision-sport athletes, to minimize bias and errors in each pre-processing stage, because the magnitudes of changes are often subtle, and brains of this age bracket are rapidly growing^46–49^. Such studies may benefit from using an unbiased brain atlas created from their study cohort, as opposed to normalizing brains of adolescent collision-sport athletes to an atlas generated from adults or healthy adolescents.

Therefore, the purpose of this work is to develop population-specific brain atlases for early-to-middle adolescent (EMA) collision-sport athletes. Based on the Purdue Neurotrauma Group (PNG) longitudinal MRI database^50^, we aim to develop:

1. One T1 template, based on the images from 215 EMA collision-sport athletes,
2. T1-based semantic labels of cortical and white matter parcellations, and
3. One DTI template, based on 64 EMA football athletes in a single competition season^51, 52^.

Regarding evaluation of the templates, our hypothesis is that compared to using a non-specific template, the PNG templates can reduce potential bias when normalizing brain images of local adolescent athletes and improve the statistical power in detecting small differences in population studies using local datasets. The evaluation includes voxel-based morphometry that characterizes the extent of shape changes of the T1 images during spatial normalization, and sensitivity of detecting longitudinal DTI changes in high school football athletes over a single season, which has been reported in previous work^51, 52^ that utilized the standardized FMRIB58 template. The brain atlases have been made available for download^53^.

## Results

### Atlas construction

**Figure 1:**
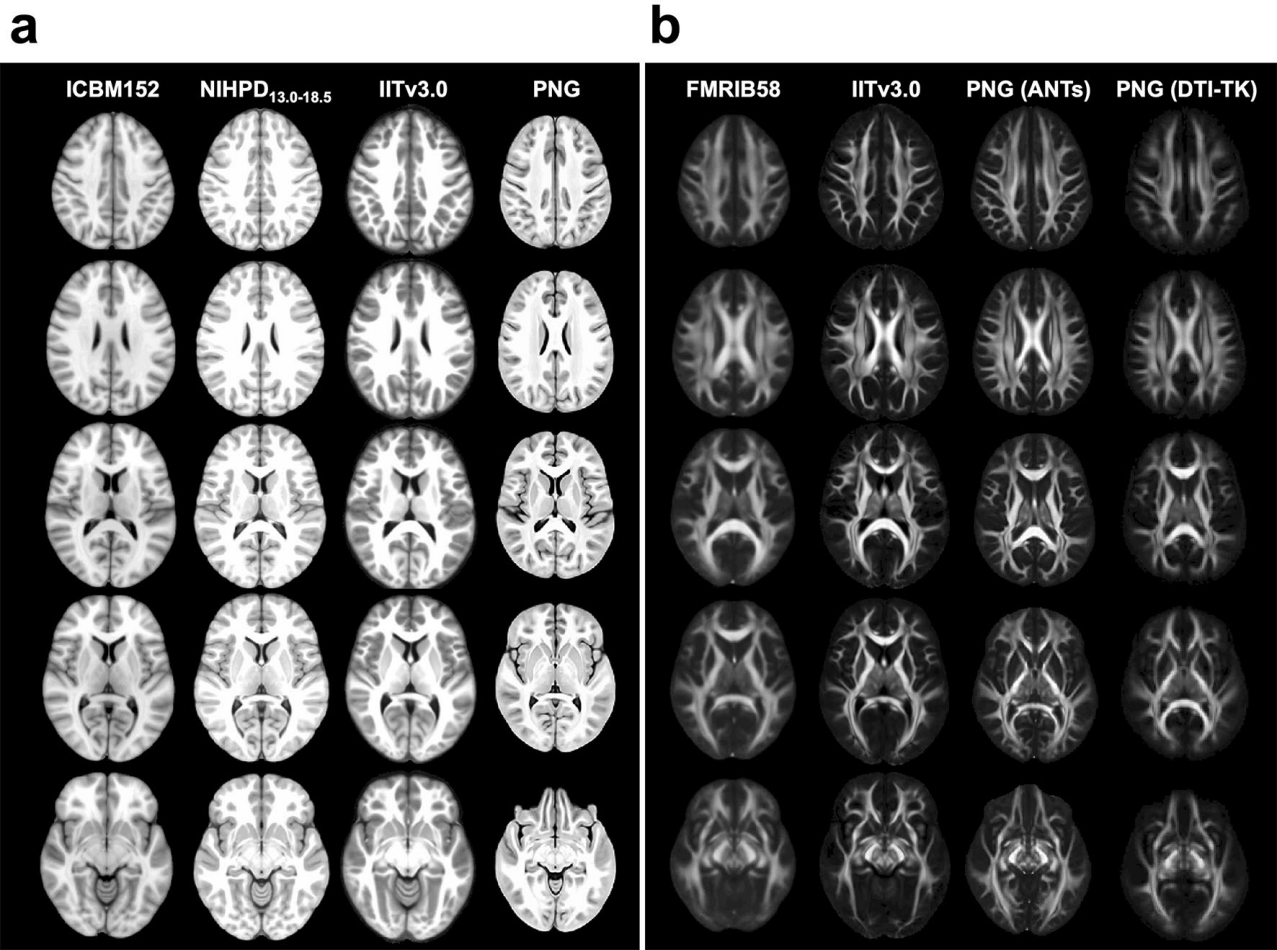
Visual illustration of the differences in shape and size between the standardized and population-specific templates, including (**a**) the standardized ICBM152 (ages 18.5–43.5)^9^, NIHPD_13.0–18.5_^21^, IITv3.0^11^, and PNG T1 templates; (**b**) the standardized FMRIB58 (FMRIB, Oxford, UK), IITv3.0^11^, and PNG DTI templates constructed by ANTs^57^ and DTI-TK^95^.

The total computation time for constructing the PNG T1 template was about 28.5 h, where ~ 6.5 h accounted for creating each individual template at the Open Science Grid, and 22 h for creating the final T1 template when fully using one node (24 cores) of the high-performance computing clusters. The comparison of shape and size between the standardized and PNG T1 templates is shown in Fig. 1a.

The total computation time of constructing the PNG (ANTs) DTI template was about 12.5 h; warping each of the *Pre* FA images (*N* = 33, with qualified T1) to the space of the PNG T1 template took ~ 2 min at the Open Science Grid, and the majority of time was spent on constructing the final DTI template when fully using one node (24 cores). The comparison of shape and size between the standardized and PNG DTI templates is shown in Fig. 1b.

### Evaluation of the population-specific T1 template

**Figure 2:**
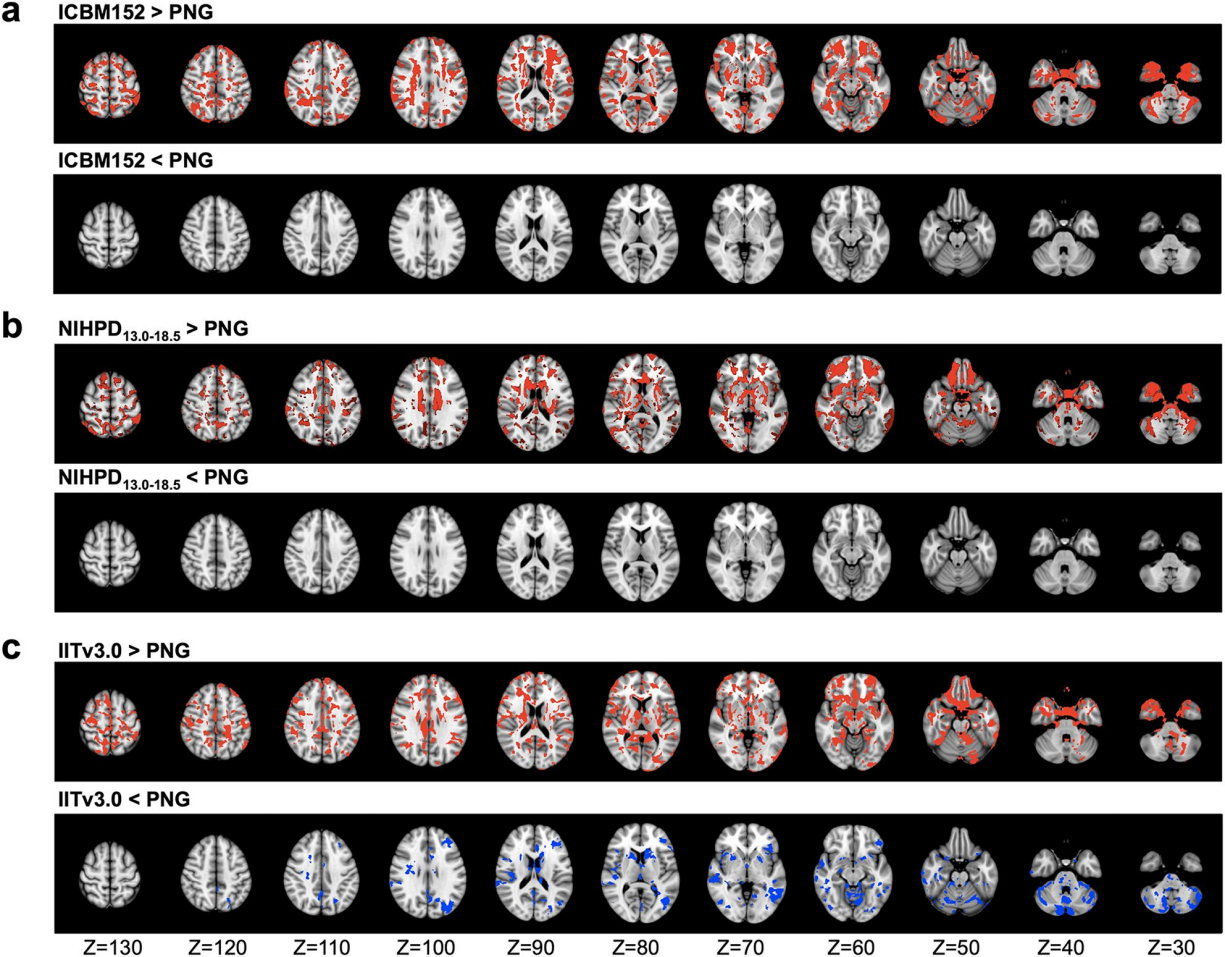
Voxel-wise *t*-statistical maps (*p* < 0.05, FWE corrected) of the deformation (|lo*gJ|*) of the newly scanned subjects, compared between the PNG T1 template and (**a**) ICBM152 (ages 18.5–43.5)^9^, (**b**) NIHPD_13.0–18.5_^21^, and (**c**) IITv3.0^11^, represented in axial view in ICBM152 space. Red/blue indicates significantly larger/smaller changes of morphology during spatial normalization, compared to using the PNG template.

The results of deformation-based morphometry analyses are shown in Fig. 2. Compared to the ICBM152 (Fig. 2a) or NIHPD_13.0–18.5_ template (Fig. 2b), no significantly larger |log*J*| was produced from using the PNG template for the spatial normalization. Compared to IITv3.0 (Fig. 2c), fewer voxels showed significantly larger |log*J*| when using the PNG template (IITv3.0: 334,811 voxels; PNG: 109,189 voxels).

### Evaluation of the study-specific DTI template

Similar to the deformation-based morphometry findings for T1 templates, at all the sessions (*Pre*, *In*1, *In*2), fewer voxels showed significantly larger |log*J*| when using the PNG (ANTs) template compared to the high-quality standardized DTI templates (FMRIB58 and IITv3.0) (Table 1). Using the PNG (ANTs) template also exhibited fewer voxels of significantly larger |log*J*| than the PNG (DTI-TK) template (Table 1). Illustrations of the voxel-wise *t*-statistical maps are provided in Supplementary Fig. S1 online.

**Table 1:** Number of statistically significant (*p* < 0.05, FWE corrected) voxels of potential bias when normalizing the FA maps of 64 high school varsity football athletes to the PNG (ANTs) DTI template and to the other templates.

Contrast	*Pre*	*In*1	*In*2
FMRIB58 > PNG (ANTs)	588,188	594,116	591,845
FMRIB58 < PNG (ANTs)	126,667	85,782	153,490
IITv3.0 > PNG (ANTs)	597,688	598,457	608,754
IITv3.0 < PNG (ANTs)	69,359	49,774	81,651
PNG (DTI-TK) > PNG (ANTs)	481,365	464,482	445,434
PNG (DTI-TK) < PNG (ANTs)	172,137	181,668	189,488

Comparisons at multiple sessions (*Pre*, *In*1, *In*2) are presented. For illustrations of the voxel-wise *t*-statistical maps, see Supplementary Fig. S1 online.

Table 2 summarizes the number of statistically significant voxels of DTI metrics (FA, MD, AD, RD) and the total number of voxels on the TBSS skeleton. The non-parametric Friedman test did not suggest the covariate template as a significant factor (${\chi }^{2}$=2.370, *df* = 3, *p* = 0.499) for ${V}_{t}$.

**Table 2:** Summary of the ratios of the number of statistically significant voxels of DTI metrics (FA, MD, AD, RD) and the total number of voxels on TBSS skeleton.

DTI	Contrast	Total significant voxels/total voxels			
		FMRIB58	IITv3.0	PNG (ANTs)	PNG (DTI-TK)
FA	*Pre* > *In*2	16,885/30,017	16,040/29,481	16,785/29,740	15,864/29,888
MD	*Pre* < *In*2	7544/30,017	6791/29,481	6838/29,740	6675/29,888
AD	*Pre* > *In*2	888/30,017	0/29,481	571/29,740	100/29,888
RD	*Pre* < *In*2	12,235/30,017	11,598/29,481	12,093/29,740	11,889/29,888

For details with respect to each ROI, see Supplementary Tables S1-S4 online.

The Hosmer Lemeshow Goodness-of-Fit test showed a good fit for the logistic regression models of FA, MD, and AD (all *p* > 0.05), except RD (*p* < 0.05) (Table 3). However, in all four models, the covariate template was a significant factor (*p* < 0.05, Wald Chi-square test) for the ${{V}_{s}/V}_{t}$ ratios (Table 3). Therefore indicating a strong correlation between the ${{V}_{s}/V}_{t}$ ratios and template selection.

**Table 3:** Summary of Hosmer Lemeshow Goodness-of-Fit test and logistic analysis for the effect of the selected template on the ratio of the number of significant voxels (from the permutation-based *t*-statistical maps) and total number of voxels on the TBSS skeleton within the ROI (see Supplementary Tables S1-S4 online).

DTI	Contrast	Goodness-of-fit test				Wald test	
		${\chi }^{2}$	*df*	*p*	${\chi }_{\mathrm{template}}^{2}$	*Df*_template_	*P*_template_
FA	*Pre* > *In*2	11.478	8	0.176	9.759	3	0.020
MD	*Pre* < *In*2	7.516	7	0.377	12.249	3	0.007
AD	*Pre* > *In*2	1.725	1	0.189	299.374	3	< 0.001
RD	*Pre* < *In*2	52.032	8	< 0.001	20.680	3	< 0.001

**Figure 3:**
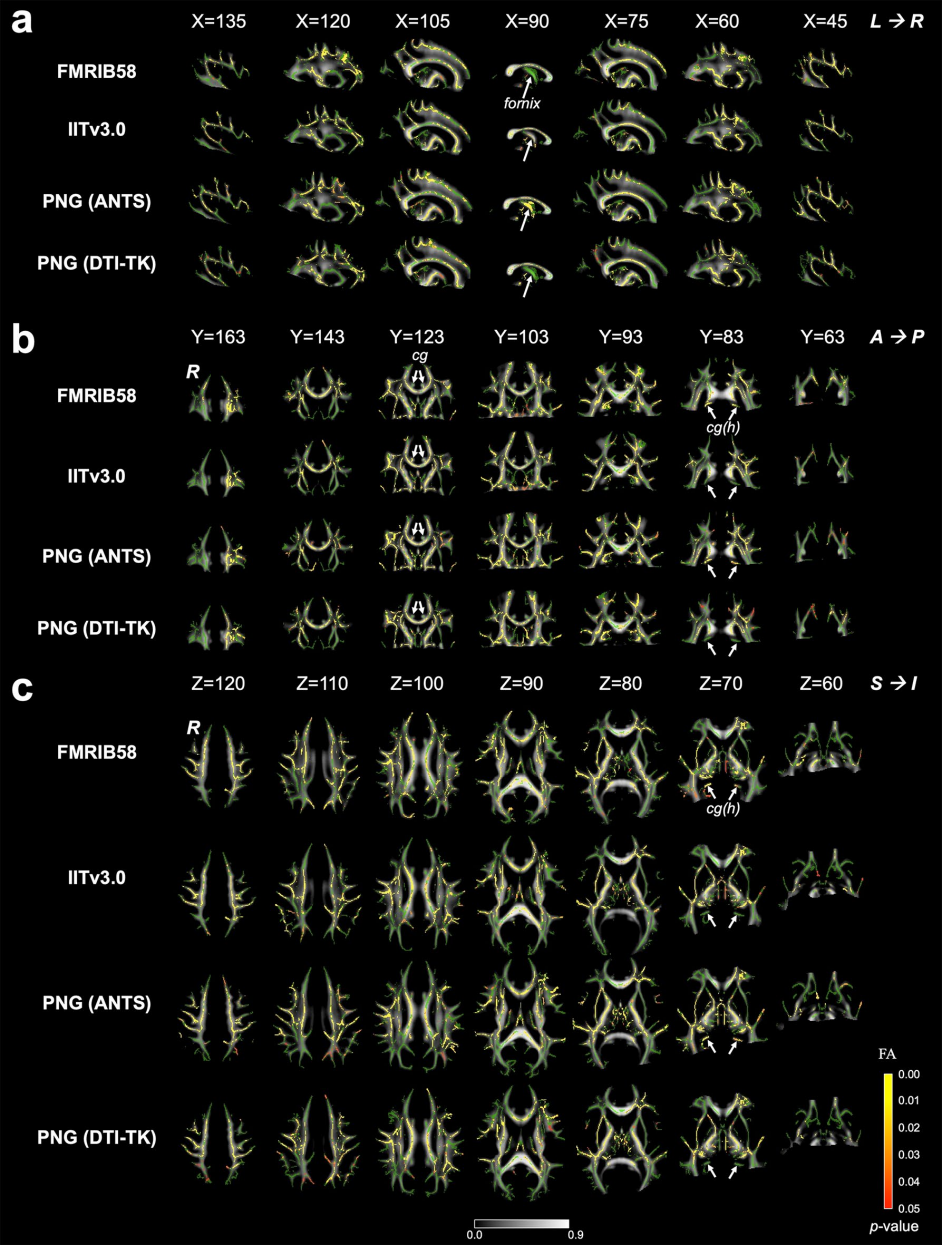
Illustrations of *t*-statistical maps (red–yellow, FWE corrected, *p* < 0.05) showing decreased FA at *In*2 versus *Pre* in (**a**) sagittal, (**b**) coronal, and (**c**) axial views, overlaid on TBSS skeleton (green) and mean FA image derived from FMRIB58 (FMRIB, Oxford, UK), IITv3.0^11^, PNG (ANTs), and PNG (DTI-TK) DTI templates respectively. Major white matter tracts showing different sensitivity across the templates for detecting FA changes are highlighted in arrows. *cg*: cingula. *cg(h)*: cingula (hippocampi). *L/R*: left/right hemisphere. *A/P*: anterior/posterior. *S/I*: superior/inferior.

Within most of the white matter tracts, the number of significant voxels exhibiting decreased FA at *In*2 versus *Pre* was similar across the four templates (Fig. 3). For the PNG (ANTs) template, the significant voxels in the fornix were 99 mm^3^, much larger compared to the FMRIB58 (14 mm^3^), IITv3.0 (5 mm^3^), and the PNG (DTI-TK) templates (13 mm^3^) (Fig. 3a). For the PNG (DTI-TK) template, the significant voxels in the bilateral cingula were much smaller compared to the other three templates (Fig. 3b). In the bilateral cingula (hippocampi), neither the IITv3.0 nor PNG (DTI-TK) template exhibited any significant voxels of FA difference (Fig. 3b,c). Similarly, no significant voxels of FA difference were observed in the left tapetum for the FMRIB58 template.

Supplementary Figs. S2-S4 online provide illustrations of the *t*-statistical maps of significant differences of MD, AD, and RD, at *In*2 versus *Pre*. A detailed summary of the significant voxels exhibiting differences of FA, MD, AD, and RD can be found in Supplementary Tables S1-S4 online.

According to the linear mixed-effect regression analyses for the longitudinal changes of FA (Supplementary Table S5 online), model fits were similar among the regions commonly identified across the four templates. FMRIB58 exhibited the highest model fit (i.e., lowest AIC) in most of the white matter tracts, including the left cerebral peduncle, left posterior corona radiata, right superior corona radiata, right external capsule, left anterior internal capsule, right posterior internal capsule, left inferior longitudinal/fronto-occipital fasciculus, and left stria terminalis. For IITv3.0, the highest fits were observed in the right cingulum, right anterior corona radiata, and right anterior internal capsule. For the PNG (ANTs) population-specific template, higher fits were observed in the bilateral superior fronto-occipital fasciculus and the right superior longitudinal fasciculus. whereas the PNG (DTI-TK) template exhibited the highest fit in the right anterior corona radiata and the left superior corona radiata.

Supplementary Tables S6 and S7 online summarize the linear mixed-effect regression analyses for the longitudinal changes, for MD and RD respectively. Similar to FA, model fits were similar among the commonly identified regions. No table is shown for AD, since no voxels exhibited a significant difference when the IITv3.0 DTI template was used (see Table 2 and Supplementary Fig. S3 online).

## Discussion

Due to repetitive head impacts experienced during practices and games, EMA collision-sport athletes may exhibit a distinct neurological trajectory that is different from those typical at the same age. Bias may be introduced when modeling the neurological consequences using the existing standardized human brain atlases based on healthy adult or adolescent populations, leading to confounding (sometimes even contradictory) findings. In this work, population-specific brain atlases were developed for EMA collision-sport athletes in the PNG longitudinal database. Compared to the standardized adult or other age-appropriate T1 templates (Fig. 1), significantly fewer biases were introduced in spatial normalization using the PNG T1 template (Fig. 2). The PNG (ANTs) DTI template resulted in minimal biases compared to the standardized or PNG (DTI-TK) DTI templates (Table 1, Supplementary Fig. S1 online), and the selection contributed to different sensitivity of detecting DTI changes in TBSS (Table 3, Fig. 3, and Supplementary Tables S1-S4 & Figs. S2-S4 online), whereas the sensitivity of detecting longitudinal change of DTI metrics from ROI-based regression analyses was relatively comparable (Supplementary Tables S5-S7 online). In summary, the main findings suggested the PNG brain atlases better characterized the neuroanatomy of EMA collision-sport athletes, reduced biases introduced during spatial normalization, and exhibited higher sensitivity in detecting regional DTI differences. As template selection is a critical strategic step towards robust and rigorous statistical findings, we expect neuroimaging and clinical researchers will benefit from the new atlases to better clarify mechanisms of mTBI and monitor brain health of EMA collision-sport athletes.

The strengths and limitations between standardized and population-specific brain atlases have been discussed^5, 6^. Being a pragmatic option for computational efficiency, a standardized brain atlas often comes with a comprehensive set of templates and semantic labels, facilitating the processing and analysis of brain images acquired from multiple sites or studies^6^. However, when the underlying neuroanatomy of the study population is different, mis-registration can lead to greater bias and errors in voxel-wise and ROI-based statistical analyses. On the other hand, the registration errors of using a population-specific template are unbiased towards the study population; however, the population-specific template usually lacks semantic labels^5^; therefore, subsequent transformation to a standard space (e.g., ICBM152) is required for interpreting the statistical maps^54^. In addition, suboptimal data quality can lead to a poorly constructed template and lowers spatial normalization accuracy^6, 11, 55^, so a nontrivial amount of diligence is demanded in constructing population-specific templates. The selection strategy largely depends on the specific study, including research questions to address, participants of the study, as well as the number, type, and quality of data^5^. Neuroimaging researchers working on clinical populations should carefully leverage these aspects to ensure rigorous and robust neuroimaging findings are reported in clinical literature and be cautious when reporting voxel-wise statistical findings based off of a non-specific brain atlas.

This work clarifies the advantages and limitations of constructing population-specific DTI templates (Fig. 1b) using scalar-based (ANTs) and tensor-based (DTI-TK) registrations. Conventionally, spatial normalization of diffusion tensor fields is achieved by aligning the *b*_0_ image to the anatomical T1 image^56^, and our evaluation showed that this approach introduced minimal biases in spatial normalization (Table 1, Supplementary Fig. S1 online). ANTs is a diffeomorphic registration that uses cross-correlation metrics to optimize the shape and appearance during template construction, with the underlying assumption that possibly different shapes of the same structures exist in both images^57^; as a result, the PNG (ANTs) template has a sharp appearance that can discern adjacent white matter tracts. Unlike ANTs, DTI-TK utilizes the six tensor components and does not include such template update procedures, and the PNG (DTI-TK) template was computed as the average of the co-registered dataset. Although the appearance was more blurred than the PNG (ANTs) template, adjacent white matter tracts can be discerned. While it is commonly believed that tensor-based registration algorithms improve the registration quality of DTI^54, 58–60^, the estimates of the tensor in certain biological structures (e.g., the fornix) can be inaccurate, which may adversely affect the quality of the constructed template. A combination of tensor information with a scalar-based registration method can potentially improve the quality of a population-specific template^61^.

**Figure 4:**
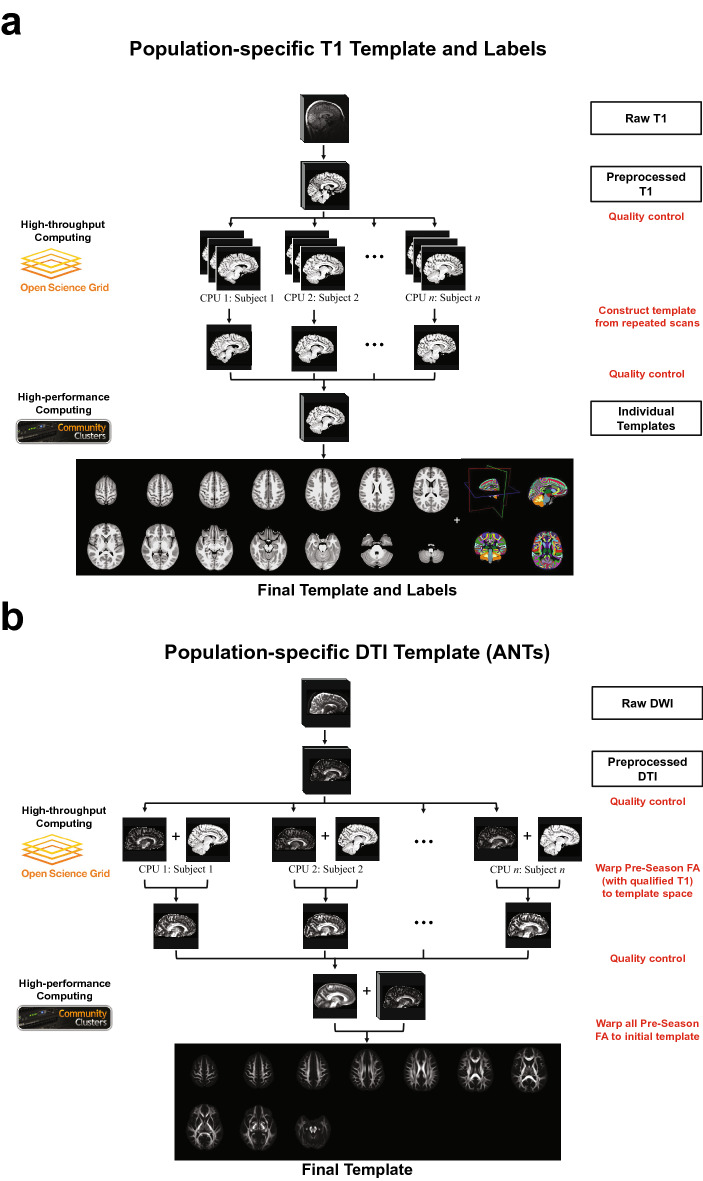
High-throughput high-performance computing workflow, for constructing (**a**) the population-specific T1 template and labels, and (**b**) the population-specific DTI template using Advanced Normalization Tools (ANTs)^57^.

According to the voxel-based morphometric analyses (Fig. 2), using the PNG T1 template introduced minimal bias during spatial normalization of the T1 images from the EMA collision-sport athletes, even when compared to the NIHPD_13.0–18.5_ template (Fig. 2b). Given the NIHPD template was constructed based on healthy adolescents of a similar age range, one explanation is that the trajectory of subcortical volumes in adolescent collision-sport athletes may be different from healthy adolescents of similar ages. Previously, Narvacan et al.^16^ reported in a lifespan study of healthy adolescents that at age 13–17, a non-linear decrease of subcortical volumes was observed within certain regions for the male participants. Thus, it is worth exploring whether the trajectory of subcortical volumes can be driven by sports-related concussion and repetitive head impacts. Notably, future work is needed to validate the T1-based semantic labels (Fig. 4a), which may be applied to investigate the regional volumetric trajectory.

The selection of DTI templates did not lead to significantly different TBSS skeleton but was a significant covariate for the voxel-wise statistical analyses (Table 2, Table 3, Supplementary Tables S1-S4 online). Previously, utilizing the standardized FMRIB58 template in TBSS processing, abnormal DTI changes in 64 adolescent football athletes from *Pre* to *In*2 were observed, including decreased FA, decreased AD, increased MD, and increased RD^51, 52^. In this work, compared to FMRIB58, the PNG (ANTs) template resulted in consistent but more sensitive detection of FA decrease within the bilateral cingula adjacent to the hippocampi [cg(h)] [PNG (ANTs): 73%, FMRIB58: 65%, IITv3.0: 3%] and the fornix [PNG (ANTs): 61%, FMRIB58: 9%, IITv3.0: 62%] (Supplementary Table S1 online), whereas on the skeletons of IITv3.0 and PNG (DTI-TK), such a difference was either detected with fewer voxels or not statistically significant (Fig. 3). The fornix and cg(h) are major parts of the limbic system. Surrounded by cerebrospinal fluids, the main body of the fornix is located in the midline of the brain, with neuronal projections to the cg(h) in medial temporal lobes. The fornix is critical for normal cognitive functioning; literature reported atrophy in the fornix for neurological disorders^62^. The cg(h) consist of gray matter, with a thin layer of white matter on its ventricular surface^63^. Atrophy of the hippocampus is a common neuropathology in chronic traumatic encephalopathy^64, 65^. The volume of the cg(h) correlated with FA in the fornix^66^, and reduced fornix and hippocampal volumes have been reported in morphometric study of TBI^67^ (for review, see Shenton et al.^68^). Both structures are relatively small and prone to mis-registration in DWI, due to low spatial resolution, geometric distortions from eddy current, and partial volume effects^69–72^. There are multiple literature that observed changes in FA for adolescent collision-sport athletes in the fornix and cingulum (hippocampus); however, the direction of change is not consistent across literature as described in our previous work^39^. Myer et al.^35^ and Kuzminski et al.^40^ reported decreased FA in cingulum (hippocampus), whereas Bazarian et al.^73^ and Manning et al.^74^ reported increased FA. Similarly, both increased^75^ and decreased FA^40^ were observed in the fornix. The conflicting direction of changes in other DTI metrics (e.g. MD, AD, RD) were also observed in the fornix^38^ and cingulum (hippocampus)^44, 74, 76^. The PNG (ANTs) template potentially provided DTI insights at improved sensitivity and complemented the volumetric findings, which is likely due to repetitive head impacts experienced by this vulnerable population. Consistent with Cabeen et al.^13^ that the study template resulted in significantly larger number of voxels in the fornix, the higher sensitivity within these areas was perhaps attributable to the better anatomical alignment in the PNG T1 template, which is unbiased towards the study population and better captured anatomical structure than the standardized templates (Fig. 1).

In addition to fornix and cingulum (hippocampus), we observed noticeably more voxels exhibiting decreased FA for the PNG (ANTs) DTI template than the standardized FMRIB58 and IITv3.0 templates, within the right cingulum (cingulate gyrus) [PNG (ANTs): 43%, FMRIB58: 8%, IITv3.0: 42%], bilateral retrolenticular internal capsules [PNG (ANTs): 76%, FMRIB58: 70%, IITv3.0: 66%], right superior longitudinal fasciculus [PNG (ANTs): 61%, FMRIB58: 55%, IITv3.0: 51%], and bilateral tapetum [PNG (ANTs): 42%, FMRIB58: 23%, IITv3.0: 35%] (Supplementary Table S1 online). Moreover, the proportions of significant voxels with decreased FA between the bilateral cingula (cingulate gyrus) are more laterality (L: 36%, R: 43%) for the PNG (ANTs) DTI template than the FMRIB58 template (L: 45%, R: 8%) (Supplementary Table S1 online). These observations may suggest that improper selection of template in neuroimage data processing can contribute to potential bias in specific ROIs.

This work has several limitations. Considering that our recruited participants were exclusively male football or female soccer players (Table 4), we constructed brain atlases only for the combined collision-sport population and not for each sport, but sex differences may exist and warrant future investigation to determine the necessity of having sex-specific brain atlases for collision sports. The visual quality assessment used in the study can be subjective and biased; future work will involve an automated and quantitative quality assessment with more specific criteria in ground truth and landmark (e.g., in Jang et al.^39^). Template selection is only one out of many aspects in the image processing pipeline that contributes to the inconsistent DTI findings reported in the mTBI literature; to achieve reproducible and meaningful results, variability in study design, scanning parameters, and analytic techniques should also be considered^45, 77, 78^. The PNG T1 template was constructed using *buildtemplateparallel.sh*, which was found to have an issue of rigid-only registration and later superseded by *antsMultivariateTemplateConstruction.sh*. This could have an impact on the quality of individual templates based on repeated scans (Fig. 4a). There are several limitations when using ANTs for template construction^5^: first, due to the differences in acquisition protocols, DWI data often have artifacts such as distortions due to eddy currents, so that mis-registration can occur between *b*_0_ and T1-weighted images. Second, since white matter has rather homogeneous intensity on a T1 image, using the warping of T1 images to guide DTI alignment may lead to a mismatch of white matter alignment. This work evaluated the proposed DTI templates using real data and compared to previous findings utilizing a standardized template^51, 52^, but a more robust scheme of evaluation is to employ simulated data, with a priori knowledge of the pathology as a ground truth^79^; such a scheme is robust for modeling pathology like multiple sclerosis, where white matter degeneration is well characterized by the corresponding FA reduction^80, 81^, but is difficult for sports-related mTBI and repetitive head impacts in adolescents, given the conflicting DTI characterization for axonal pathology in the literature^45^.

**Table 4:** Summary of the datasets for constructing and evaluating the population-specific T1 and DTI templates, based on the Purdue Neurotrauma Group (PNG) longitudinal MRI database^50^.

Task	Season	Session^a^	Sport^b^ (n)	Age range (mean ± std)	n Ethnicity (FB, SOC)
**PNG T1 template**					
Construction	2011–2017	*Pre In Post*	FB (155) SOC (60)	All: 13–19 (16.16 ± 1.05) FB: 13–19 (16.20 ± 1.08) SOC: 14–18 (16.04 ± 0.97)	White: 157 (104, 53) Black or African American: 21 (21, 0) Hispanic or Latino: 11 (8, 3) Asian: 2 (2, 0) Native American: 5 (5, 0) More than one: 17 (13, 4) Unspecified: 2 (2, 0)
Evaluation	2018–2019	*Pre*	FB (12)	14–19 (16.67 ± 1.37)	White: 6 Black or African American: 2 Hispanic or Latino: 1 Native American: 1 More than one: 2
**PNG DTI template**					
Construction	2016–2017	*Pre*	FB (64)	14–18 (16.00 ± 1.04)	White: 36 Black or African American: 9 Hispanic or Latino: 5 Native American: 3 More than one: 7 Unspecified: 4
Evaluation^c^	2016–2017	*Pre* *In*			

^a^Data were acquired across multiple sessions, including one approximately one month before contact practices began (*Pre*), one or more within competition season (*In*), and one or more after the season ended (*Post*).

^b^All football athletes (FB) are male participants, and all soccer athletes (SOC) are female participants.

^c^Same sport, age range, and ethnicity as the dataset for constructing the DTI template.

## Methods

### Participants and data collection

This study used data collected by PNG in their ongoing longitudinal study of adolescent athletes^50^, which has been approved by the Biomedical IRB of Purdue’s Human Research Protection Program and was carried out in accordance with the Declaration of Helsinki. Before enrolling in the study, written informed consent was obtained from each participant, and subject assent and parental consent were obtained for participants under the age of 18. The data include athletes participating in the collision sports of American football (all males) and soccer (all females). Data were acquired across multiple sessions, including one approximately one month before contact practices began (*Pre*), one or more within competition season (*In*), and one or more after the season ended (*Post*). These data were grouped into different datasets to construct or evaluate the population-specific T1 or DTI templates. See Table 4 for the total number of participants and relevant details for each dataset.

Note that during the period of study, no participant was diagnosed by their athletic trainer or team physician as being concussed.

### MR imaging

All data were acquired using a 3 T General Electric Signa HDx (Waukesha, WI) with a 16-channel brain array (Nova Medical; Wilmington, MA).

#### T1-weighted imaging data

Anatomical T1 scans were acquired using a 3D fast spoiled gradient-echo sequence (TR/TE = 5.7/2.0 ms, tip angle = 73°, 1 mm isotropic resolution). Longitudinal volumetric data from 227 athletes (167 males; 60 females) were used for construction and evaluation of the template (Table 4).

#### Diffusion-weighted imaging data

Diffusion-weighted imaging (DWI) data were acquired using a spin-echo echo-planar imaging sequence (TR/TE = 12,500/100 ms, 40 slices with 2.5 mm thickness), FOV of 24 × 24 cm^2^, a 96 × 96 acquisition matrix, in-plane resolution of 2.5 × 2.5 mm^2^, with 30 diffusion-encoding directions at b = 1000 s/mm^2^ and one at b = 0 s/mm^2^, and an upsampled isotropic resolution of 1 mm. Longitudinal data were from sixty-four male football athletes that participated in one competition season (Table 4). All participants completed three MRI sessions: one scan at *Pre* and two In-Season scans (*In*), with one in the first (*In*1) and one in the second (*In*2) 5-week halves of the season.

### Atlas construction

To accelerate the computation time, we established a workflow integrating a high-throughput (The Open Science Grid)^82, 83^ and a high-performance (Purdue Community Clusters) computing platform. Specifically, the Open Science Grid integrates the computing and storage elements from over 100 individual sites spanning the United States and provides a distributed fabric of high-throughput computational services, allowing numerous individual, small, and independent tasks to run concurrently on different CPU cores. Purdue Community Clusters consists of Dell compute nodes with 16–24 cores of Intel Xeon Gold Sky Lake processors per node, at least 192 GB of RAM, and 100 Gbps InfiniBand interconnects, which processes single, large, and interdependent tasks at its fastest speed. The workflow was implemented to construct the population-specific T1 template and one of the two population-specific DTI templates, and the schematic diagrams were shown in Fig. 4.

#### Population-specific T1 template and labels

The population consisted of 215 EMA collision-sport athletes (ages: 13 − 19, 16.16 ± 1.05) scanned at *Pre* of the 2011–2017 competition seasons, including 155 high school varsity football athletes (ages: 13 − 19, 16.20 ± 1.08) and 60 soccer athletes (ages: 14 − 18, 16.04 ± 0.97) (Table 4).

The workflow of constructing the PNG (ANTs) template is summarized in Fig. 4a. T1 preprocessing included (1) denoising using an adaptive non-local (NL)-means filter to improve signal-to-noise ratio across all the spatial-frequency domains of the image^84^; (2) bias correction using FSL FAST to correct for spatial intensity variations and minimize the influence of intensity gradient on segmentation^85, 86^; (3) skull-stripping using a brain extraction program to remove non-brain part of the image (*FSL BET*)^87, 88^; and (4) intensity normalization using FSL MATH commands (*fslmaths -inm*) to make T1 images between subject comparable^89^, followed by the first visual quality assessment where preprocessed T1 images with low signals, cutoff of brain regions, motion, or observable artifacts were excluded; this resulted in 782 T1 images from 235 participants, where 547 were repeated scans from 168 participants.

Advanced Normalization Tools (ANTs)^57^, a top-performing registration tool, was applied to construct the T1 template. ANTs employs symmetric groupwise normalization that has been shown to retain accurate anatomical details^90^. Specifically, ANTs utilizes Symmetric Normalization (SyN)^91^ as the registration algorithm, and formulate the problem of atlas construction as “estimating a common space and set of transformations that gives the smallest parameterization of the dataset.” The size and shape of the template image is optimized to converge to the group mean via Symmetric Groupwise Normalization (SyGN)^92^, which is achieved mathematically by optimizing

$${E}_{\stackrel{-}{I}}={\sum }_{i}{E}_{{\text{SyN}},\prod }\left(\stackrel{-}{I},{J}^{i},{\phi }^{i}\right),\mathrm{where} \forall i, {\phi }^{i}\left(x,0\right)=\psi (x)$$

where $\stackrel{-}{I}$ is the template image, ${J}^{i}$ is the *i*th individual image, ${\phi }^{i}$ is the diffeomorphism, where the initial conditions of each ${\phi }^{i}$ is denoted as $\psi$. For complete explanations of the algorithm, see Avants et al.^57, 92^.

Using *buildtemplateparallel.sh* in ANTs, one individual template was created per participant on the Open Science Grid, with 30 × 50 × 20 iterations per registration, and cross correlation as the evaluation metric (*buildtemplateparallel.sh -d 3 -m* 30 × 50 × 20 *-t GR -s CC -c 1*), followed by a second visual quality assessment, which mainly focused on resemblance of neuroanatomy pertinent to each individual, including the matching of major sulci and gyri, and gray matter-CSF border; this resulted in individual templates from 215 participants with good quality. Using Purdue Community Clusters, the final population-specific template (PNG T1) was created from the individual templates, with one full node (24 cores) and one indivitual template as the target volume (*buildtemplateparallel.sh -d 3 -m 30* × *50* × *20 -t GR -s CC -c 2 -j 24 -z*).

Based on the final template, the semantic labels were created using the *recon-all* pipeline of FreeSurfer (v6.0.0)^93^, employing the Desikan-Killiany labeling protocol^94^ to assign the neuroanatomical label to each cortical region. The template and labels have been made available at the Purdue University Research Repository^53^.

### Population-specific DTI templates

The population consisted of 64 football athletes (age: 14–18, 16.00 ± 1.04) scanned at *Pre* of the 2016–2017 competition season (Table 4).

Two top-performing registration tools, namely ANTs^57^ and DTI-TK^95^, was applied to construct DTI templates. DTI-TK incorporates explicit optimization of tensor orientation with piecewise affine registration^95^, and the problem of atlas construction is formulated as “estimating an image that requires the minimum amount of deformation to map into every image in the population.”^96^ Given a population of *N* diffusion tensor images ${\left\{{J}^{i}\right\}}_{i=1}^{N}$, the template estimation is defined mathmatically as^97^.

$$\left\{{\widehat{H}}_{i}, \widehat{J}\right\}=\underset{{H}_{i}, J}{\mathrm{argmin}}\sum_{i=1}^{N}\left({\int }_{{\mathbb{R}}^{3}}^{ }{\Vert {J}^{i}{\circ H}_{i}\left(\mathbf{x}\right)-J\left(\mathbf{x}\right)\Vert }^{2}d\mathbf{x}+D\left({H}_{i}\right)\right),$$

where ${H}_{i}$ is the deformation applied to the image ${J}^{i}$. The tensor metric $\Vert \cdot \Vert$ represents the image term, which is the summation of the region-wise tensor image difference. $D({H}_{i})$ is a metric that quantifies the amount of deformation associated with ${H}_{i}$. For complete explanations of the algorithm, see Zhang et al.^95, 97^.

Before constructing the templates, raw DWI data were first preprocessed using FSL (FMRIB 5.0, Oxford, U.K.), including corrections for motion and eddy currents (*eddy_correct*), followed by the extraction of aliasing-corrected brains (*BET*). DTI metrics, including FA, mean diffusivity (MD), axial diffusivity (AD), and radial diffusivity (RD), were estimated for each individual (*DTIFit*), and all passed the first visual quality assessment for presence of motion artifact or geometrical distortion.

The workflow of constructing the PNG (ANTs) template is summarized in Fig. 4b. Based on the quality assessments when constructing the population-specific T1 template, 33 of the 64 football players had a qualified T1 image at *Pre*. Only the corresponding DWI images at *Pre* were used, considering that DTI changes were observed at *In*1 and *In*2^51, 52^. For each subject, the *b*_0_ image served as the reference image to warp the FA image to the corresponding T1 image, and subsequently to the PNG T1 template space. All warping processes were carried out by running *antsIntermodalityIntrasubject.sh*. All the warped FA images passed the second visual quality assessment for inspecting whether they were normalized to the same space of the template. Then, an average map of the warped FA images was computed to serve as the initial reference image to register to, and the population-specific DTI template was constructed based on the 64 football players at *Pre*, with one full node (24 cores) and one indivitual template as the target volume (*antsMultivariateTemplateConstruction.sh -d 3 -m 30x50x20 -t GR -s CC -c 2 -j 24 -i 4 -z*), and has been made available at the Purdue University Research Repository^53^. The second DTI template was constructed using DTI-TK^95^ as a comparison to the PNG (ANTs) template, where the diffusion tensors in the native space of 64 subjects were used.

## Evaluations

### Population-specific T1 template

The population consisted of 12 newly scanned high school varsity football athletes (ages: 14–19, 16.67 ± 1.37) scanned at *Pre* of the 2018–2019 competition season (Table 4).

Deformation-based morphometry analyses were performed to compare the potential bias of using different T1 templates. The newly acquired T1 scans were normalized (via *antsRegistrationSyN*) to the ICBM152 template, an age-appropriate template (NIHPD_13.0–18.5_^21^, IITv3.0^11^, and the population-specific T1 template (PNG); this yielded 4 × 12 = 48 maps of deformation field. The logarithm of Jacobian determinant (log*J*, representing local volume difference) was estimated (via *ANTSJacobian*) for each map. The maps of absolute log*J* (|log*J*|) were computed and transformed to the standard space of ICBM152 (1 mm spatial resolution) via *antsApplyTransforms*. In the standard space, voxel-wise permutation-based *t*-statistics were computed with 5000 permutations with a repeated ANOVA design, using the FSL Randomise program^98^, with threshold-free cluster enhancement^99^ and family wise error (FWE) of 5% used to control for type-I error.

### Population-specific DTI templates

The population consisted of 64 high school varsity football athletes (ages: 14–18, 16.00 ± 1.04) scanned at *Pre, In*1, and *In*2 of the 2016–2017 competition season (Table 4).

All individual FA images were first aligned through a nonlinear transformation algorithm (*FNIRT*) to four DTI templates, including two standardized templates: FMRIB58 (FMRIB, Oxford, UK) and IITv3.0^11^, and the two PNG population-specific templates constructed by ANTs and DTI-TK.

Similar to evaluating the T1 templates, deformation-based morphometry analyses were performed to compare the potential bias of using different DTI templates. At each session (*Pre*, *In*1, *In*2), 4 × 64 = 256 maps of |log*J*| were yielded, and all were transformed to the standard space of FMRIB58 (same as ICBM152). Using the same design as evaluating the T1 templates, voxel-wise permutation-based *t*-statistics were computed for each session.

In the standard space of ICBM152, a skeleton representing the common white matter tracts across all the subjects was created from thinning the mean FA map that was averaged from all the aligned FA images. The skeleton was thresholded at FA > 0.2 to reduce partial volume effects between borders of different tissues. Regional maximal FA values were projected onto the skeleton according to a distance map^27^. Based on the mean FA skeleton, skeletons of MD, AD, and RD were obtained by projecting the corresponding DTI values onto the FA skeleton (*tbss_non_FA*). The processing procedure guaranteed that the variations of the TBSS results were only related to the template selection.

The resulting DTI skeletons of each subject were fed into voxel-wise permutation-based statistics with a repeated ANOVA design and with 5000 permutations among *Pre*, *In*1 and *In*2, using the FSL Randomise program^98^. The type-I error was controlled by threshold-free cluster enhancement^99^ and FWE of 5%. For the purpose of demonstrating the effect of template selection on subsequent statistical findings, only the contrasts comparing *Pre* and *In*2 were presented. Among all the selected contrasts of each template, the ones showing significant voxels at *p* < 0.05 (FA and AD: *Pre* > *In*2; MD and RD: *Pre* < *In*2) were further segmented into ROIs defined by the JHU-ICBM-DTI-81 WM label atlas^100^, and the corresponding voxel counts and DTI values were extracted via the FSL Cluster program.

Within each ROI overlaid on the TBSS skeletons, we counted

$${V}_{t}=\mathrm{The\ number\ of\ voxels\ on\ the\ skeleton},$$

and

$${V}_{s}=\mathrm{The\ number\ of\ significant\ voxels\ from\ the\ permutation\mbox{-}based\ statistics.}$$

First, the non-parametric Friedman test was performed to test whether ${V}_{t}$ correlated with template selection, with ${V}_{t}$ as the response variable, template as the predictor, and ROI as the blocking variable.

Then, logistic regression was performed to test whether ${{V}_{s}/V}_{t}$ correlated with template selection:

$$\mathrm{log}\frac{{p}_{ij}}{1-{p}_{ij}}={\beta }_{0}+{\beta }_{1}\times {\text{Template}}_{i}+{\beta }_{2}\times {\text{ROI}}_{j}$$

where ${p}_{ij}$ referred to the ${{V}_{s}/V}_{t}$ ratio from the *i*th template, with regard to the TBSS skeleton within the *j*th ROI. 4 models were established with respect to the ${{V}_{s}/V}_{t}$ ratio of each DTI metric (FA, MD, AD and RD). ROIs with no voxel on the TBSS skeletons were excluded from the analyses. The analyses were performed using SAS 9.4 (SAS Institute, Cary NC).

To investigate the sensitivity of different templates to the short-term changes of white matter microstructure in high-school football players^51, 52^, linear mixed regression analyses were performed, where timepoint and age were the fixed variables, and subject was the random variable. Models were fitted within each ROI and for each DTI metric. The Akaike information criterion (AIC) was used to evaluate model fit, and *t* and *p* values for timepoint were compared across the four templates. FDR was applied to correct for comparisons in multiple ROIs. ROIs with no voxel on the skeleton, and ROIs rejected by the Shapiro-Wilks normality test were excluded from the analyses. The analyses were performed using R version 3.5.2^101^.

## Supplementary Information

Supplementary Information

## Data Availability

https://doi.org/10.4231/RTXE-0Q70.
