# Supplementary material for: Development of brain atlases for early-to-middle adolescent collision-sport athletes
Source: Sci Rep. 2021 Mar 19;11:6440. doi: 10.1038/s41598-021-85518-6 (PMC7979742; doi:10.1038/s41598-021-85518-6)
Supplement: Supplementary file 1 — Supplementary Information [file 41598_2021_85518_MOESM1_ESM.pdf]

Title: Development of brain atlases for early-to-middle adolescent collision-sport athletes

Full Author List: Yukai Zou[1,2], Wenbin Zhu[3], Ho-Ching (Shawn) Yang[1], Ikbeom Jang[4], Nicole L. Vike[5], Diana O. Svaldi[1], Trey E. Shenk[4], Victoria N. Poole[1,5], Evan L. Breedlove[6], Gregory G. Tamer, Jr.[1], Larry J. Leverenz[7]; Ulrike Dydak[8], Eric A. Nauman[1,5,6], Yunjie Tong[1], Thomas M. Talavage[1,4] and Joseph V. Rispoli[1,4,*]

Institutional Affiliations:

[1]Weldon School of Biomedical Engineering, Purdue University, West Lafayette, IN, USA

[2]College of Veterinary Medicine, Purdue University, West Lafayette, IN, USA

[3]Department of Statistics, Purdue University, West Lafayette, IN, USA

[4]School of Electrical and Computer Engineering, Purdue University, West Lafayette, IN, USA

[5]Department of Basic Medical Sciences, Purdue University, West Lafayette, IN, USA

[6]School of Mechanical Engineering, Purdue University, West Lafayette, IN, USA

[7]Department of Health and Kinesiology, Purdue University, West Lafayette, IN, USA

[8]School of Health Sciences, Purdue University, West Lafayette, IN, USA

[*] **Corresponding Author:** Joseph V. Rispoli, 206 S Martin Jischke Drive, West Lafayette, IN 47907-2032, U.S.A., Phone: +1 (765) 494-6178, Fax: +1 (765) 496-1459, E-mail: jrispoli@purdue.edu

Supplementary Figures and Tables

Supplementary Fig. S1 Voxel-wise *t*-statistical maps (p<0.05, corrected) of potential bias, comparing PNG (ANTs) template to high-quality standardized DTI templates (FMRIB58 and IITv3.0), and to PNG (DTI-TK) template, represented in axial view in ICBM152 space. Red/blue indicates significantly larger/smaller changes of morphology during spatial normalization, compared to using PNG (ANTs) template.

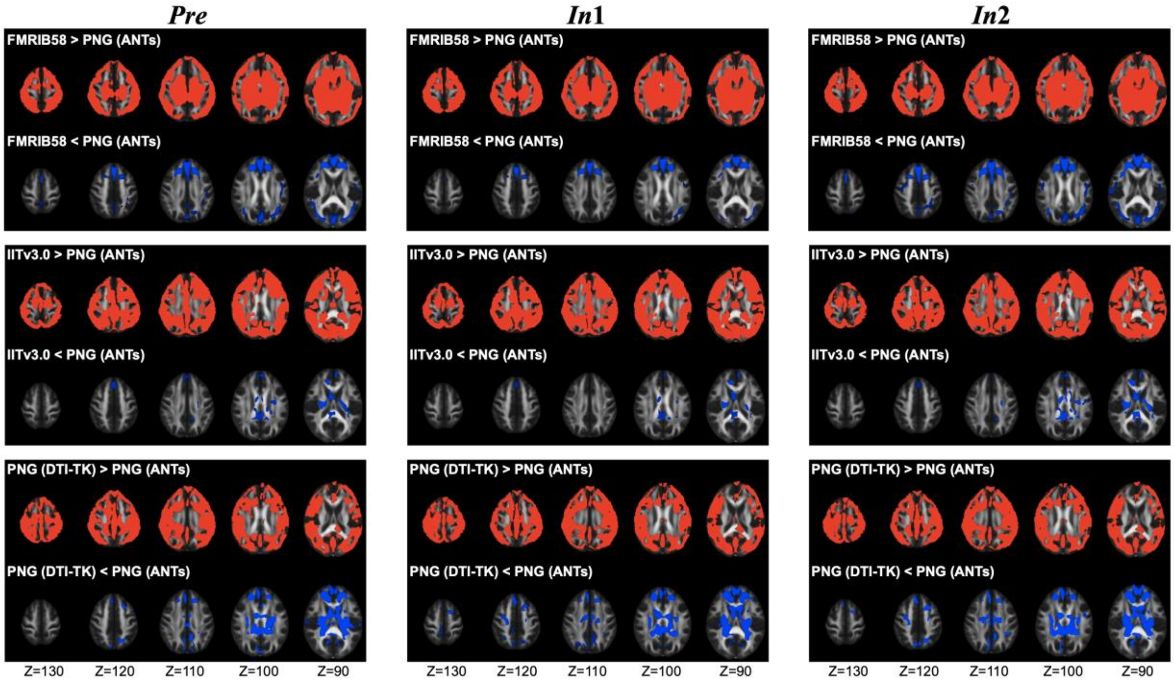

Supplementary Fig. S2 Illustrations of *t*-statistical maps (blue-lightblue, FWE corrected, *p*<0.05) showing increased MD at *In*2 vs. *Pre* in sagittal, coronal, and axial views, overlaid on TBSS skeleton (green) and mean FA image derived from FMRIB58, IITv3.0, PNG (ANTs), and PNG (DTI-TK) DTI templates respectively. *L/R*: left/right hemisphere. *A/P*: anterior/posterior. *S/I*: superior/inferior.

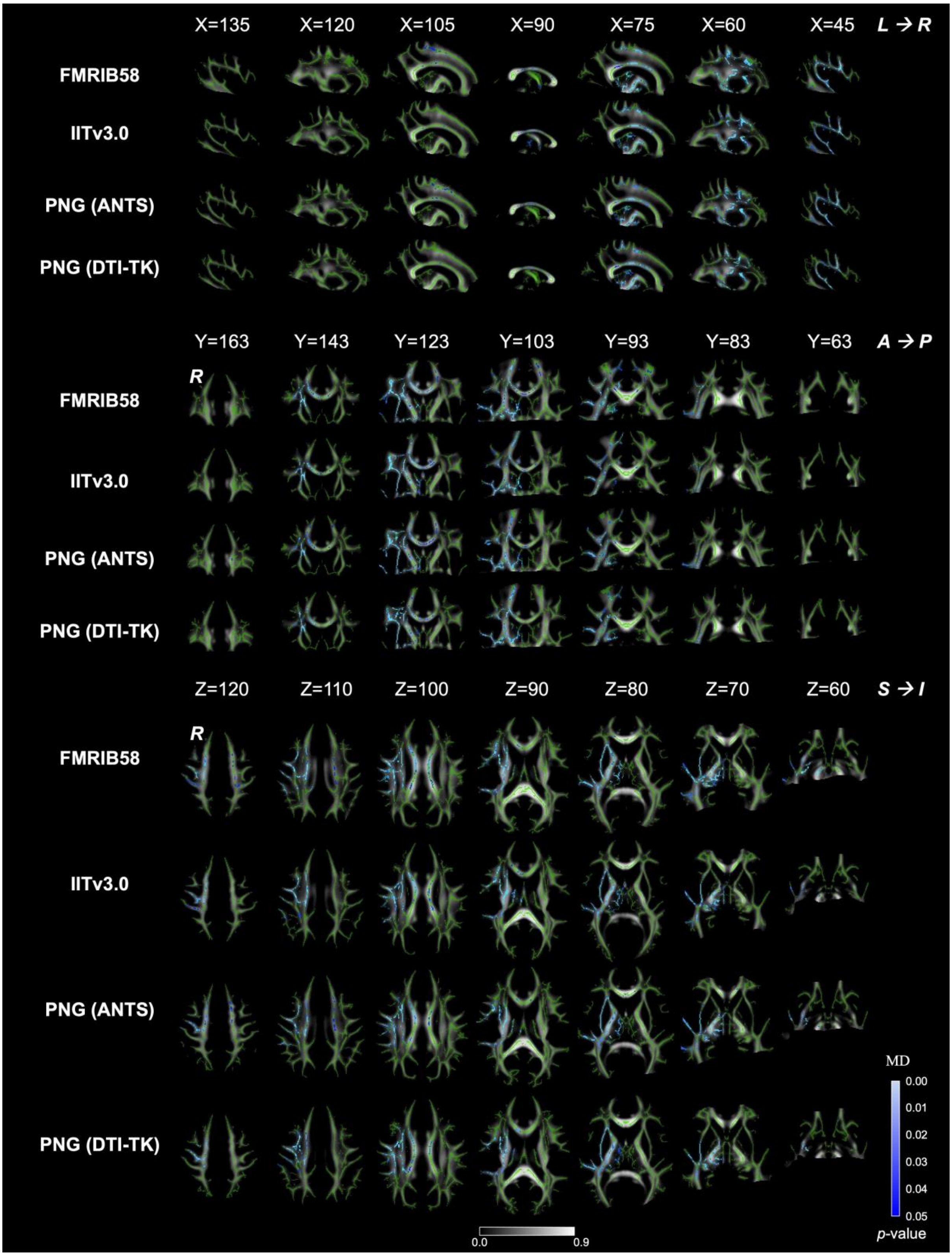

Supplementary Fig. S3 Illustrations of *t*-statistical maps (red-yellow, FWE corrected, $p<0.05$) showing decreased AD at *In*2 vs. *Pre* in sagittal, coronal, and axial views, overlaid on TBSS skeleton (green) and mean FA image derived from FMRIB58, IITv3.0, PNG (ANTs), and PNG (DTI-TK) DTI templates respectively. *L/R*: left/right hemisphere. *A/P*: anterior/posterior. *S/I*: superior/inferior.

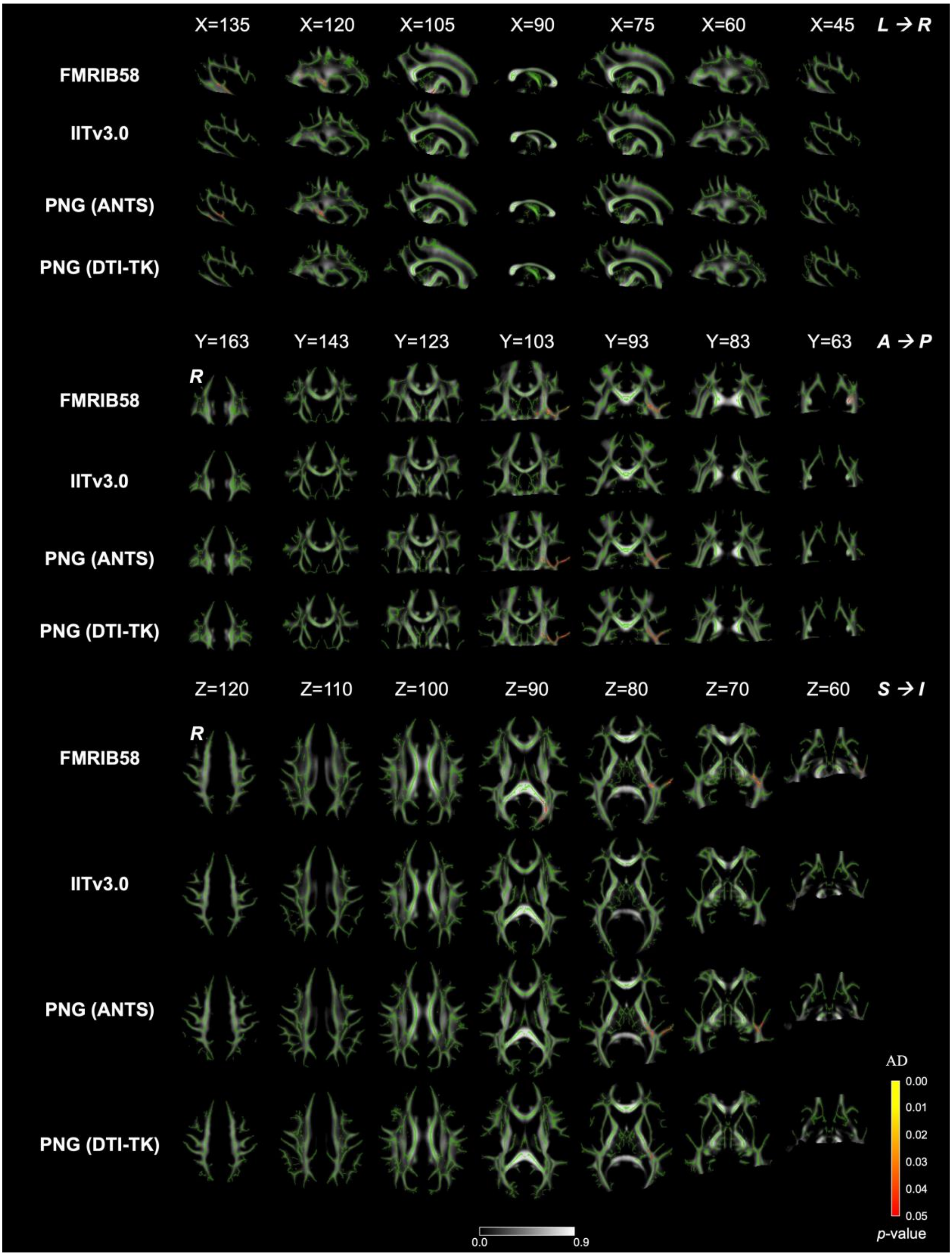

Supplementary Fig. S4 Illustrations of *t*-statistical maps (blue-lightblue, FWE corrected, *p*<0.05) showing increased RD at *In*2 vs. *Pre* in sagittal, coronal, and axial views, overlaid on TBSS skeleton (green) and mean FA image derived from FMRIB58, IITv3.0, PNG (ANTs), and PNG (DTI-TK) DTI templates respectively. *L/R*: left/right hemisphere. *A/P*: anterior/posterior. *S/I*: superior/inferior.

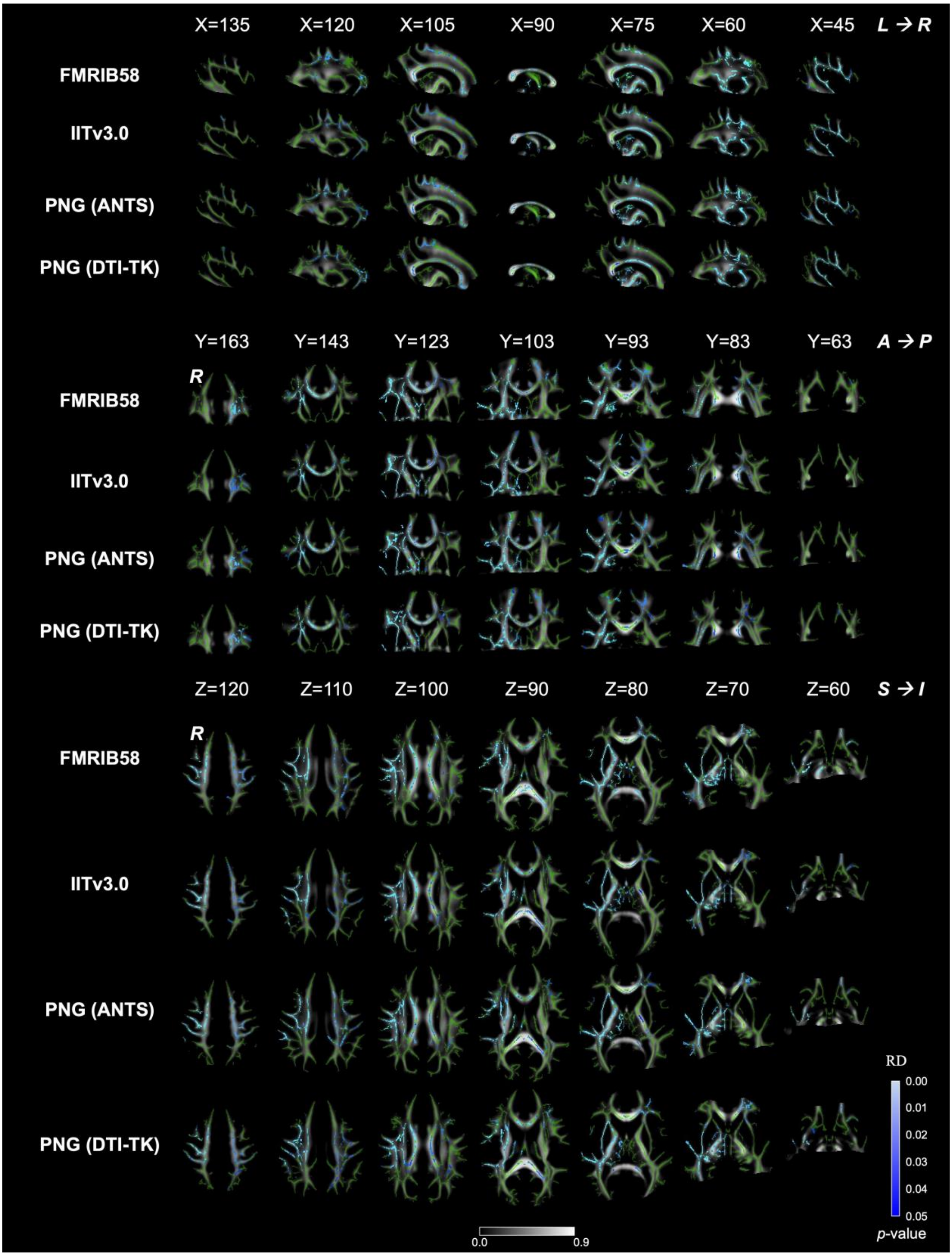

Abbreviations in Supplementary Tables S1-S4:

acr	Corona radiata - anterior
alic	Internal capsule - anterior
bcc	Corpus callosum - body
cg	Cingulum (cingulate gyrus)
cg(h)	Cingulum (hippocampus)
cp	Cerebral peduncle
ec	External capsule
gcc	Corpus callosum - genu
Ilf/Ifof	Inferiorongitudinal/fronto-occipital fasciculus
pcr	Corona radiata - posterior
plic	Internal capsule - posterior
ptr	Posterior thalamic radiation
rlic	Internal capsule - retrolenticular
scc	Corpus callosum - splenium
scr	Corona radiata - superior
sfof	Superior fronto-occipital fasciculus
slf	Superior longitudinal fasciculus
st	Stria terminalis
uf	Uncinate fasciculus

Supplementary Table S1 Summary of the number of statistically significant voxels that exhibit lower FA at *In*2 vs. *Pre* (numerator), with respect to the corresponding total number of voxels within each ROI (denominator), for the standardized and population-specific DTI templates. L/R: Left/right hemisphere.

ROI	FMRIB58	IITv3.0	PNG (ANTs)	PNG (DTI-TK)
cp L	188/247	178/230	199/244	185/240
cp R	230/283	189/249	229/271	205/253
cg L	221/487	126/278	116/320	5/213
cg R	32/422	106/252	108/251	6/111
scr L	841/1234	892/1318	932/1370	946/1345
scr R	1182/1323	1225/1369	1260/1387	1200/1363
acr L	874/1546	868/1583	828/1583	836/1589
acr R	499/1504	414/1628	374/1635	368/1633
pcr L	425/741	381/695	319/683	285/687
pcr R	324/818	292/820	278/859	257/819
bcc	2057/3142	2180/3182	2256/3295	2188/3360
gcc	907/1743	935/1823	933/1696	878/1786
scc	1717/2528	1389/2278	1383/2397	1321/2397
ec L	166/1353	123/1351	93/1287	102/1340
ec R	858/1239	809/1240	850/1162	818/1244
fornix	14/162	88/142	99/162	13/168
cg(h) L	25/60	2/14	24/42	0/53
cg(h) R	86/110	0/44	63/77	0/81
ilf/Ifof L	96/241	75/183	91/185	87/205
ilf/Ifof R	270/334	186/279	205/258	161/225
alic L	381/776	355/703	337/729	345/735
alic R	550/786	554/771	509/725	536/760
plic L	576/859	641/853	650/857	703/862
plic R	690/858	659/835	713/855	744/872
rlic L	468/782	421/796	525/795	525/793
rlic R	615/767	611/774	664/768	639/759
ptr L	265/991	182/881	174/755	152/763
ptr R	484/1101	192/969	309/912	155/872
st L	221/316	133/174	185/249	177/239
st R	274/288	129/132	221/227	216/223
sfof L	28/93	57/114	52/117	52/112
sfof R	62/98	83/112	88/113	95/112
slf L	478/1332	714/1694	640/1662	716/1769
slf R	765/1402	833/1640	1030/1702	892/1760
tapetum L	0/2	10/15	20/29	30/54
tapetum R	5/20	3/22	15/54	19/78
uf L	0/16	0/28	0/10	0/6
uf R	11/13	5/10	13/17	7/7
**Total**	**16885/30017**	**16040/29481**	**16785/29740**	**15864/29888**

Supplementary Table S2 Summary of the number of statistically significant voxels that exhibit higher MD at *In*2 vs. *Pre* (numerator), with respect to the corresponding total number of voxels within each ROI (denominator), for the standardized and population-specific DTI templates. L/R: Left/right hemisphere.

ROI	FMRIB58	IITv3.0	PNG (ANTs)	PNG (DTI-TK)
cp L	0/247	0/230	0/244	0/240
cp R	185/283	135/249	168/271	143/253
cg L	0/487	22/278	0/320	0/213
cg R	216/422	99/252	0/251	0/111
scr L	137/1234	5/1318	107/1370	5/1345
scr R	1014/1323	1089/1369	1072/1387	1098/1363
acr L	0/1546	0/1583	7/1583	0/1589
acr R	309/1504	322/1628	294/1635	290/1633
pcr L	8/741	0/695	0/683	0/687
pcr R	204/818	170/820	168/859	186/819
bcc	1225/3142	1104/3182	955/3295	1025/3360
gcc	312/1743	154/1823	324/1696	186/1786
scc	73/2528	22/2278	53/2397	52/2397
ec L	0/1353	0/1351	0/1287	0/1340
ec R	843/1239	842/1240	821/1162	805/1244
fornix	0/162	44/142	2/162	0/168
cg(h) L	0/60	0/14	0/42	0/53
cg(h) R	0/110	0/44	0/77	0/81
ilf/Ifof L	0/241	0/183	0/185	0/205
ilf/Ifof R	229/334	169/279	178/258	152/225
alic L	0/776	0/703	0/729	0/735
alic R	518/786	465/771	460/725	473/760
plic L	0/859	0/853	0/857	0/862
plic R	703/858	669/835	680/855	725/872
rlic L	0/782	0/796	0/795	0/793
rlic R	409/767	385/774	376/768	362/759
ptr L	0/991	0/881	0/755	0/763
ptr R	123/1101	103/969	93/912	99/872
st L	0/316	0/174	0/249	0/239
st R	180/288	63/132	136/227	130/223
sfof L	0/93	0/114	0/117	0/112
sfof R	78/98	99/112	93/113	76/112
slf L	0/1332	0/1694	0/1662	0/1769
slf R	762/1402	821/1640	834/1702	861/1760
tapetum L	0/2	0/15	0/29	0/54
tapetum R	3/20	0/22	0/54	0/78
uf L	0/16	0/28	0/10	0/6
uf R	13/13	9/10	17/17	7/7
**Total**	**7544/30017**	**6791/29481**	**6838/29740**	**6675/29888**

Supplementary Table S3 Summary of the number of statistically significant voxels that exhibit lower AD at *In*2 vs. *Pre* (numerator), with respect to the corresponding total number of voxels within each ROI (denominator), for the standardized and population-specific DTI templates. L/R: Left/right hemisphere.

ROI	FMRIB58	IITv3.0	PNG (ANTs)	PNG (DTI-TK)
cp L	109/247	0/230	0/244	0/240
cp R	0/283	0/249	0/271	0/253
cg L	0/487	0/278	0/320	0/213
cg R	0/422	0/252	0/251	0/111
scr L	0/1234	0/1318	0/1370	0/1345
scr R	0/1323	0/1369	0/1387	0/1363
acr L	0/1546	0/1583	0/1583	0/1589
acr R	0/1504	0/1628	0/1635	0/1633
pcr L	35/741	0/695	0/683	0/687
pcr R	0/818	0/820	0/859	0/819
bcc	0/3142	0/3182	0/3295	0/3360
gcc	0/1743	0/1823	0/1696	0/1786
scc	67/2528	0/2278	0/2397	0/2397
ec L	63/1353	0/1351	40/1287	0/1340
ec R	0/1239	0/1240	0/1162	0/1244
fornix	0/162	0/142	0/162	0/168
cg(h) L	0/60	0/14	0/42	0/53
cg(h) R	0/110	0/44	0/77	0/81
ilf/Ifof L	57/241	0/183	75/185	0/205
ilf/Ifof R	0/334	0/279	0/258	0/225
alic L	0/776	0/703	0/729	0/735
alic R	0/786	0/771	0/725	0/760
plic L	48/859	0/853	23/857	0/862
plic R	0/858	0/835	0/855	0/872
rlic L	389/782	0/796	402/795	95/793
rlic R	0/767	0/774	0/768	0/759
ptr L	100/991	0/881	26/755	5/763
ptr R	0/1101	0/969	0/912	0/872
st L	16/316	0/174	5/249	0/239
st R	0/288	0/132	0/227	0/223
sfof L	0/93	0/114	0/117	0/112
sfof R	0/98	0/112	0/113	0/112
slf L	0/1332	0/1694	0/1662	0/1769
slf R	0/1402	0/1640	0/1702	0/1760
tapetum L	0/2	0/15	0/29	0/54
tapetum R	0/20	0/22	0/54	0/78
uf L	4/16	0/28	0/10	0/6
uf R	0/13	0/10	0/17	0/7
**Total**	**888/30017**	**0/29481**	**571/29740**	**100/29888**

Supplementary Table S4 Summary of the number of statistically significant voxels that exhibit higher RD at *In*2 vs. *Pre* (numerator), with respect to the corresponding total number of voxels within each ROI (denominator), for the standardized and population-specific DTI templates. L/R: Left/right hemisphere.

ROI	FMRIB58	IITv3.0	PNG (ANTs)	PNG (DTI-TK)
cp L	0/247	0/230	0/244	0/240
cp R	237/283	185/249	223/271	192/253
cg L	229/487	121/278	139/320	52/213
cg R	227/422	112/252	125/251	4/111
scr L	569/1546	513/1583	445/1583	597/1589
scr R	419/1504	391/1628	404/1635	407/1633
acr L	122/741	105/695	127/683	111/687
acr R	236/818	260/820	256/859	244/819
pcr L	373/1234	457/1318	302/1370	511/1345
pcr R	1206/1323	1285/1369	1296/1387	1249/1363
bcc	1792/3142	1804/3182	1873/3295	1901/3360
gcc	690/1743	702/1823	677/1696	689/1786
scc	1227/2528	1024/2278	1099/2397	1017/2397
ec L	39/1353	34/1351	0/1287	22/1340
ec R	933/1239	943/1240	929/1162	959/1244
fornix	19/162	75/142	7/162	0/168
cg(h) L	0/60	0/14	0/42	0/53
cg(h) R	0/110	0/44	0/77	0/81
ilf/Ifof L	0/241	0/183	0/185	0/205
ilf/Ifof R	282/334	204/279	205/258	175/225
alic L	0/776	0/703	0/729	0/735
alic R	583/786	563/771	532/725	539/760
plic L	0/859	80/853	111/857	0/862
plic R	719/858	707/835	727/855	773/872
rlic L	67/782	0/796	124/795	2/793
rlic R	584/767	571/774	622/768	600/759
ptr L	112/991	53/881	64/755	3/763
ptr R	197/1101	220/969	157/912	214/872
st L	0/316	0/174	0/249	0/239
st R	260/288	118/132	209/227	199/223
sfof L	0/93	0/114	0/117	0/112
sfof R	87/98	107/112	105/113	99/112
slf L	169/1332	69/1694	250/1662	295/1769
slf R	840/1402	877/1640	1046/1702	1001/1760
tapetum L	0/2	5/15	9/29	11/54
tapetum R	4/20	3/22	13/54	16/78
uf L	0/16	0/28	0/10	0/6
uf R	13/13	10/10	17/17	7/7
**Total**	**12235/30017**	**11598/29481**	**12093/29740**	**11889/29888**

Supplementary Table S5 Summary of linear mixed-effect regression analyses of the longitudinal FA changes during a single season. L/R: Left/right hemisphere.

ROI	Template	AIC	Timepoint		FA (mean ± SE)
			t	*p*	
Cerebral peduncle L	FMRIB58	-999.01	-3.933	<0.001	$0.77 \pm 2.64\times10^{-3}$
Cerebral peduncle L	IITv3.0	-965.84	-4.022	<0.001	$0.76 \pm 2.86\times10^{-3}$
Cerebral peduncle L	PNG (ANTs)	-973.71	-3.951	<0.001	$0.77 \pm 2.89\times10^{-3}$
Cerebral peduncle L	PNG (DTI-TK)	-957.59	-3.832	<0.001	$0.77 \pm 2.95\times10^{-3}$
Cingulum R	FMRIB58	-700.25	-2.424	0.017	$0.52 \pm 6.56\times10^{-3}$
Cingulum R	IITv3.0	-842.96	-3.613	<0.001	$0.60 \pm 4.14\times10^{-3}$
Cingulum R	PNG (ANTs)	-807.77	-3.089	0.002	$0.58 \pm 4.80\times10^{-3}$
Cingulum R	PNG (DTI-TK)	-503.29	-1.590	0.114	$0.51 \pm 9.82\times10^{-3}$
Corona radiata - anterior L	FMRIB58	-949.87	-5.608	<0.001	$0.54 \pm 4.32\times10^{-3}$
Corona radiata - anterior L	IITv3.0	-965.64	-5.730	<0.001	$0.52 \pm 4.10\times10^{-3}$
Corona radiata - anterior L	PNG (ANTs)	-950.56	-5.427	<0.001	$0.51 \pm 4.30\times10^{-3}$
Corona radiata - anterior L	PNG (DTI-TK)	-938.73	-5.380	<0.001	$0.53 \pm 4.20\times10^{-3}$
Corona radiata - anterior R	FMRIB58	-909.23	-4.273	<0.001	$0.51 \pm 4.38\times10^{-3}$
Corona radiata - anterior R	IITv3.0	-910.47	-4.359	<0.001	$0.49 \pm 4.14\times10^{-3}$
Corona radiata - anterior R	PNG (ANTs)	-885.24	-3.761	<0.001	$0.48 \pm 4.23\times10^{-3}$
Corona radiata - anterior R	PNG (DTI-TK)	-915.48	-3.729	<0.001	$0.47 \pm 3.81\times10^{-3}$
Corona radiata - posterior L	FMRIB58	-984.76	-3.561	0.001	$0.52 \pm 3.44\times10^{-3}$
Corona radiata - posterior L	IITv3.0	-936.43	-3.208	0.002	$0.53 \pm 3.87\times10^{-3}$
Corona radiata - posterior L	PNG (ANTs)	-949.14	-2.919	0.004	$0.53 \pm 3.70\times10^{-3}$
Corona radiata - posterior L	PNG (DTI-TK)	-948.28	-3.060	0.003	$0.53 \pm 3.93\times10^{-3}$
Corona radiata - superior L	FMRIB58	-1001.89	-3.744	<0.001	$0.53 \pm 3.35\times10^{-3}$
Corona radiata - superior L	IITv3.0	-1006.36	-3.888	<0.001	$0.52 \pm 3.26\times10^{-3}$
Corona radiata - superior L	PNG (ANTs)	-1015.91	-4.134	<0.001	$0.52 \pm 3.24\times10^{-3}$
Corona radiata - superior L	PNG (DTI-TK)	-1016.41	-4.144	<0.001	$0.52 \pm 3.21\times10^{-3}$
Corona radiata - superior R	FMRIB58	-976.17	-4.211	<0.001	$5.23 \pm 3.39\times10^{-3}$
Corona radiata - superior R	IITv3.0	-974.19	-4.117	<0.001	$5.24 \pm 3.29\times10^{-3}$
Corona radiata - superior R	PNG (ANTs)	-976.06	-4.314	<0.001	$5.22 \pm 3.33\times10^{-3}$
Corona radiata - superior R	PNG (DTI-TK)	-966.94	-4.117	<0.001	$5.17 \pm 3.25\times10^{-3}$
External capsule R	FMRIB58	-978.78	-3.761	<0.001	$0.46 \pm 2.66\times10^{-3}$
External capsule R	IITv3.0	-966.54	-3.966	<0.001	$0.45 \pm 2.71\times10^{-3}$
External capsule R	PNG (ANTs)	-973.99	-3.771	<0.001	$0.44 \pm 2.68\times10^{-3}$
External capsule R	PNG (DTI-TK)	-959.33	-3.905	<0.001	$0.45 \pm 2.79\times10^{-3}$

(cont.)

ROI	Template	AIC	Timepoint		FA (mean ± SE)
			t	p	
Internal capsule - anterior L	FMRIB58	-997.92	-4.395	<0.001	$0.64 \pm 2.69\times10^{-3}$
Internal capsule - anterior L	IITv3.0	-983.46	-4.613	<0.001	$0.64 \pm 2.94\times10^{-3}$
Internal capsule - anterior L	PNG (ANTs)	-969.37	-4.228	<0.001	$0.64 \pm 2.89\times10^{-3}$
Internal capsule - anterior L	PNG (DTI-TK)	-975.60	-4.363	<0.001	$0.64 \pm 2.87\times10^{-3}$
Internal capsule - anterior R	FMRIB58	-893.71	-3.600	<0.001	$0.64 \pm 3.21\times10^{-3}$
Internal capsule - anterior R	IITv3.0	-897.37	-4.006	<0.001	$0.63 \pm 3.10\times10^{-3}$
Internal capsule - anterior R	PNG (ANTs)	-890.68	-3.790	<0.001	$0.64 \pm 3.25\times10^{-3}$
Internal capsule - anterior R	PNG (DTI-TK)	-888.37	-3.921	<0.001	$0.63 \pm 3.24\times10^{-3}$
Internal capsule - posterior R	FMRIB58	-963.365	-3.631	<0.001	$0.73 \pm 3.11\times10^{-3}$
Internal capsule - posterior R	IITv3.0	-940.976	-3.519	0.001	$0.74 \pm 3.29\times10^{-3}$
Internal capsule - posterior R	PNG (ANTs)	-936.896	-3.512	0.001	$0.75 \pm 3.40\times10^{-3}$
Internal capsule - posterior R	PNG (DTI-TK)	-934.373	-3.565	0.001	$0.75 \pm 3.39\times10^{-3}$
Ilf/Ifof L	FMRIB58	-803.62	-3.122	0.002	$0.53 \pm 4.86\times10^{-3}$
Ilf/Ifof L	IITv3.0	-756.94	-2.823	0.006	$0.52 \pm 5.31\times10^{-3}$
Ilf/Ifof L	PNG (ANTs)	-782.88	-2.949	0.004	$0.52 \pm 5.07\times10^{-3}$
Ilf/Ifof L	PNG (DTI-TK)	-773.21	-2.870	0.005	$0.52 \pm 5.20\times10^{-3}$
Stria terminalis L	FMRIB58	-762.92	-2.749	0.007	$0.59 \pm 4.19\times10^{-3}$
Stria terminalis L	IITv3.0	-779.92	-3.256	0.001	$0.60 \pm 4.20\times10^{-3}$
Stria terminalis L	PNG (ANTs)	-770.46	-2.895	0.004	$0.59 \pm 4.16\times10^{-3}$
Stria terminalis L	PNG (DTI-TK)	-758.32	-2.880	0.005	$0.59 \pm 4.27\times10^{-3}$
Superior fronto-occipital fasciculus R	FMRIB58	-815.55	-2.341	0.021	$0.50 \pm 4.38\times10^{-3}$
Superior fronto-occipital fasciculus R	IITv3.0	-828.50	-2.756	0.007	$0.53 \pm 4.14\times10^{-3}$
Superior fronto-occipital fasciculus R	PNG (ANTs)	-857.78	-2.601	0.010	$0.53 \pm 3.85\times10^{-3}$
Superior fronto-occipital fasciculus R	PNG (DTI-TK)	-800.03	-2.527	0.013	$0.51 \pm 4.60\times10^{-3}$
Superior fronto-occipital fasciculus L	FMRIB58	-867.90	-2.153	0.033	$0.52 \pm 4.15\times10^{-3}$
Superior fronto-occipital fasciculus L	IITv3.0	-875.89	-2.657	0.009	$0.57 \pm 3.56\times10^{-3}$
Superior fronto-occipital fasciculus L	PNG (ANTs)	-900.60	-2.504	0.014	$0.58 \pm 3.71\times10^{-3}$
Superior fronto-occipital fasciculus L	PNG (DTI-TK)	-854.08	-2.480	0.014	$0.56 \pm 3.91\times10^{-3}$
Superior longitudinal fasciculus L	FMRIB58	-971.10	-3.546	0.001	$0.53 \pm 3.68\times10^{-3}$
Superior longitudinal fasciculus L	IITv3.0	-996.79	-4.505	<0.001	$0.51 \pm 3.63\times10^{-3}$
Superior longitudinal fasciculus L	PNG (ANTs)	-980.14	-4.146	<0.001	$0.51 \pm 3.67\times10^{-3}$
Superior longitudinal fasciculus L	PNG (DTI-TK)	-1004.10	-4.592	<0.001	$0.50 \pm 3.62\times10^{-3}$
Superior longitudinal fasciculus R	FMRIB58	-902.52	-3.357	0.001	$0.53 \pm 3.76\times10^{-3}$
Superior longitudinal fasciculus R	IITv3.0	-882.84	-3.333	0.001	$0.51 \pm 3.78\times10^{-3}$
Superior longitudinal fasciculus R	PNG (ANTs)	-904.06	-3.397	0.001	$0.51 \pm 3.59\times10^{-3}$
Superior longitudinal fasciculus R	PNG (DTI-TK)	-892.06	-3.408	0.001	$0.50 \pm 3.60\times10^{-3}$

Supplementary Table S6 Summary of linear mixed-effect regression analyses of the longitudinal MD changes during a single season. L/R: Left/right hemisphere.

ROI	Template	AIC	Timepoint		MD (mean ± SE)
			t	*p*	
Corona radiata - superior L	FMRIB58	-3540.24	2.901	0.004	$0.76{\times}10^{-3} \pm 3.75{\times}10^{-6}$
Corona radiata - superior L	IITv3.0	-3397.20	1.620	0.108	$0.76{\times}10^{-3} \pm 5.13{\times}10^{-6}$
Corona radiata - superior L	PNG (ANTs)	-3531.50	2.907	0.004	$0.74{\times}10^{-3} \pm 3.62{\times}10^{-6}$
Corona radiata - superior L	PNG (DTI-TK)	-3379.13	1.812	0.072	$0.75{\times}10^{-3} \pm 5.19{\times}10^{-6}$
Corona radiata - posterior R	FMRIB58	-3519.73	3.933	<0.001	$0.76{\times}10^{-3} \pm 4.17{\times}10^{-6}$
Corona radiata - posterior R	IITv3.0	-3513.54	4.119	<0.001	$0.76{\times}10^{-3} \pm 4.18{\times}10^{-6}$
Corona radiata - posterior R	PNG (ANTs)	-3516.49	3.991	<0.001	$0.76{\times}10^{-3} \pm 4.21{\times}10^{-6}$
Corona radiata - posterior R	PNG (DTI-TK)	-3526.66	4.227	<0.001	$0.77{\times}10^{-3} \pm 4.17{\times}10^{-6}$
External capsule R	FMRIB58	-3442.10	3.672	<0.001	$0.79{\times}10^{-3} \pm 3.77{\times}10^{-6}$
External capsule R	IITv3.0	-3432.47	3.605	<0.001	$0.79{\times}10^{-3} \pm 3.84{\times}10^{-6}$
External capsule R	PNG (ANTs)	-3434.88	3.625	<0.001	$0.79{\times}10^{-3} \pm 3.77{\times}10^{-6}$
External capsule R	PNG (DTI-TK)	-3427.17	3.626	<0.001	$0.79{\times}10^{-3} \pm 3.85{\times}10^{-6}$
Internal capsule - anterior R	FMRIB58	-3486.41	4.128	<0.001	$0.74{\times}10^{-3} \pm 3.30{\times}10^{-6}$
Internal capsule - anterior R	IITv3.0	-3490.87	4.339	<0.001	$0.73{\times}10^{-3} \pm 3.28{\times}10^{-6}$
Internal capsule - anterior R	PNG (ANTs)	-3489.21	4.291	<0.001	$0.74{\times}10^{-3} \pm 3.30{\times}10^{-6}$
Internal capsule - anterior R	PNG (DTI-TK)	-3481.10	4.353	<0.001	$0.73{\times}10^{-3} \pm 3.36{\times}10^{-6}$
Internal capsule - retrolenticular R	FMRIB58	-3496.76	3.524	0.001	$0.78{\times}10^{-3} \pm 3.67{\times}10^{-6}$
Internal capsule - retrolenticular R	IITv3.0	-3483.14	3.648	<0.001	$0.78{\times}10^{-3} \pm 3.74{\times}10^{-6}$
Internal capsule - retrolenticular R	PNG (ANTs)	-3497.66	3.622	<0.001	$0.79{\times}10^{-3} \pm 3.56{\times}10^{-6}$
Internal capsule - retrolenticular R	PNG (DTI-TK)	-3491.50	3.481	0.001	$0.79{\times}10^{-3} \pm 3.58{\times}10^{-6}$
Posterior thalamic radiation R	FMRIB58	-3292.57	3.475	0.001	$0.78{\times}10^{-3} \pm 7.56{\times}10^{-6}$
Posterior thalamic radiation R	IITv3.0	-3286.59	3.504	0.001	$0.78{\times}10^{-3} \pm 7.47{\times}10^{-6}$
Posterior thalamic radiation R	PNG (ANTs)	-3289.02	3.581	<0.001	$0.79{\times}10^{-3} \pm 7.29{\times}10^{-6}$
Posterior thalamic radiation R	PNG (DTI-TK)	-3289.95	3.432	0.001	$0.79{\times}10^{-3} \pm 7.08{\times}10^{-6}$
Stria terminalis R	FMRIB58	-3400.11	3.900	<0.001	$0.79{\times}10^{-3} \pm 4.21{\times}10^{-6}$
Stria terminalis R	IITv3.0	-3360.82	4.016	<0.001	$0.81{\times}10^{-3} \pm 4.70{\times}10^{-6}$
Stria terminalis R	PNG (ANTs)	-3391.48	4.221	<0.001	$0.80{\times}10^{-3} \pm 4.27{\times}10^{-6}$
Stria terminalis R	PNG (DTI-TK)	-3393.78	4.121	<0.001	$0.79{\times}10^{-3} \pm 4.29{\times}10^{-6}$

Supplementary Table S7 Summary of linear mixed-effect regression analyses of the longitudinal RD changes during a single season. L/R: Left/right hemisphere.

ROI	Template	AIC	Timepoint		RD (mean ± SE)
			t	***p***	
Corona radiata - anterior L	FMRIB58	-3428.68	3.485	0.001	$0.50\times10^{-3} \pm 5.15\times10^{-6}$
Corona radiata - anterior L	IITv3.0	-3425.70	3.424	0.001	$0.50\times10^{-3} \pm 5.04\times10^{-6}$
Corona radiata - anterior L	PNG (ANTs)	-3423.19	3.378	0.001	$0.50\times10^{-3} \pm 5.17\times10^{-6}$
Corona radiata - anterior L	PNG (DTI-TK)	-3445.18	3.519	0.001	$0.50\times10^{-3} \pm 4.63\times10^{-6}$
Corona radiata - posterior L	FMRIB58	-3504.33	3.278	0.001	$0.57\times10^{-3} \pm 5.20\times10^{-6}$
Corona radiata - posterior L	IITv3.0	-3534.10	3.353	0.001	$0.58\times10^{-3} \pm 4.78\times10^{-6}$
Corona radiata - posterior L	PNG (ANTs)	-3524.54	2.962	0.004	$0.57\times10^{-3} \pm 4.82\times10^{-6}$
Corona radiata - posterior L	PNG (DTI-TK)	-3521.96	3.226	0.002	$0.56\times10^{-3} \pm 4.73\times10^{-6}$
Corona radiata - posterior R	FMRIB58	-3458.14	3.595	<0.001	$0.52\times10^{-3} \pm 5.15\times10^{-6}$
Corona radiata - posterior R	IITv3.0	-3491.90	3.634	<0.001	$0.52\times10^{-3} \pm 4.62\times10^{-6}$
Corona radiata - posterior R	PNG (ANTs)	-3478.75	3.543	0.001	$0.52\times10^{-3} \pm 4.91\times10^{-6}$
Corona radiata - posterior R	PNG (DTI-TK)	-3478.70	3.678	<0.001	$0.52\times10^{-3} \pm 5.02\times10^{-6}$
Corona radiata - superior L	FMRIB58	-3541.79	3.433	0.001	$0.51\times10^{-3} \pm 4.19\times10^{-6}$
Corona radiata - superior L	IITv3.0	-3557.26	3.209	0.002	$0.51\times10^{-3} \pm 3.73\times10^{-6}$
Corona radiata - superior L	PNG (ANTs)	-3527.16	3.132	0.002	$0.50\times10^{-3} \pm 4.28\times10^{-6}$
Corona radiata - superior L	PNG (DTI-TK)	-3573.73	3.386	0.001	$0.52\times10^{-3} \pm 3.66\times10^{-6}$
Corona radiata - superior R	FMRIB58	-3530.15	3.637	<0.001	$0.49\times10^{-3} \pm 3.53\times10^{-6}$
Corona radiata - superior R	IITv3.0	-3525.04	3.624	<0.001	$0.50\times10^{-3} \pm 3.49\times10^{-6}$
Corona radiata - superior R	PNG (ANTs)	-3520.92	3.709	<0.001	$0.50\times10^{-3} \pm 3.53\times10^{-6}$
Corona radiata - superior R	PNG (DTI-TK)	-3510.80	3.691	<0.001	$0.50\times10^{-3} \pm 3.55\times10^{-6}$
Cingulum R	FMRIB58	-3418.20	3.696	<0.001	$0.47\times10^{-3} \pm 4.67\times10^{-6}$
Cingulum R	IITv3.0	-3449.84	4.119	<0.001	$0.45\times10^{-3} \pm 4.00\times10^{-6}$
Cingulum R	PNG (ANTs)	-3409.71	3.802	<0.001	$0.46\times10^{-3} \pm 4.82\times10^{-6}$
Cingulum R	PNG (DTI-TK)	-3076.68	2.334	0.021	$0.52\times10^{-3} \pm 1.16\times10^{-5}$
External capsule R	FMRIB58	-3441.82	3.673	<0.001	$0.58\times10^{-3} \pm 3.84\times10^{-6}$
External capsule R	IITv3.0	-3430.02	3.626	<0.001	$0.59\times10^{-3} \pm 3.91\times10^{-6}$
External capsule R	PNG (ANTs)	-3435.03	3.640	<0.001	$0.59\times10^{-3} \pm 3.83\times10^{-6}$
External capsule R	PNG (DTI-TK)	-3438.87	3.679	<0.001	$0.59\times10^{-3} \pm 3.80\times10^{-6}$
Internal capsule - posterior R	FMRIB58	-3523.57	3.928	<0.001	$0.35\times10^{-3} \pm 3.54\times10^{-6}$
Internal capsule - posterior R	IITv3.0	-3505.42	3.850	<0.001	$0.34\times10^{-3} \pm 3.71\times10^{-6}$
Internal capsule - posterior R	PNG (ANTs)	-3492.87	3.860	<0.001	$0.33\times10^{-3} \pm 3.94\times10^{-6}$
Internal capsule - posterior R	PNG (DTI-TK)	-3490.18	3.804	<0.001	$0.33\times10^{-3} \pm 3.94\times10^{-6}$

(cont.)

ROI	Template	AIC	Timepoint		RD (mean ± SE)
			t	*p*	
Internal capsule - retrolenticular R	FMRIB58	-3448.23	3.659	<0.001	$0.47\times10^{-3} \pm 4.43\times10^{-6}$
Internal capsule - retrolenticular R	IITv3.0	-3444.53	3.594	<0.001	$0.48\times10^{-3} \pm 4.42\times10^{-6}$
Internal capsule - retrolenticular R	PNG (ANTs)	-3454.16	3.501	0.001	$0.48\times10^{-3} \pm 4.21\times10^{-6}$
Internal capsule - retrolenticular R	PNG (DTI-TK)	-3439.08	3.285	0.001	$0.48\times10^{-3} \pm 4.34\times10^{-6}$
Superior longitudinal fasciculus L	FMRIB58	-3546.15	3.125	0.002	$0.51\times10^{-3} \pm 3.93\times10^{-6}$
Superior longitudinal fasciculus L	IITv3.0	-3506.09	3.064	0.003	$0.51\times10^{-3} \pm 4.18\times10^{-6}$
Superior longitudinal fasciculus L	PNG (ANTs)	-3535.54	2.973	0.004	$0.51\times10^{-3} \pm 4.22\times10^{-6}$
Superior longitudinal fasciculus L	PNG (DTI-TK)	-3561.37	3.435	0.001	$0.53\times10^{-3} \pm 3.86\times10^{-6}$
